# Neurodevelopment and brain maturation in late preterm children: insights from the adolescent brain cognitive development (ABCD) study

**DOI:** 10.3389/fped.2026.1827902

**Published:** 2026-07-20

**Authors:** Ryoko Yoshinare, Masatoshi Yamashita, Yoshifumi Mizuno, Akemi Tomoda

**Affiliations:** 1 Division of Developmental Higher Brain Functions, United Graduate School of Child Development, Osaka University, Kanazawa University, Hamamatsu University School of Medicine, Chiba University, and University of Fukui, Fukui, Japan; 2 Department of Child and Adolescent Psychological Medicine, University of Fukui Hospital, Fukui, Japan; 3 Research Center for Child Mental Development, University of Fukui, Fukui, Japan; 4 Research Center for Child Mental Development, Hamamatsu University School of Medicine, Shizuoka, Japan; 5 Life Science Innovation Center, School of Medical Science, University of Fukui, Fukui, Japan

**Keywords:** adolescence, brain structure, diffusion tensor imaging, late preterm, neurodevelopment trajectory, ABCD study

## Abstract

**Introduction:**

Neurodevelopmental outcomes in late preterm (LP) children, born between 34 and 36 gestation weeks, remain unclear. This study aimed to examine behavioral, cognitive, and multimodal neuroimaging outcomes in children born late preterm compared with children born full term during early adolescence using data from the large U.S.-based Adolescent Brain Cognitive Development Study, with longitudinal mixed-effects models used as the primary analytic framework to evaluate group, timepoint, and group-by-timepoint effects.

**Methods:**

Participants aged 9–11 years at baseline were categorized into LP and full-term (FT; ≥40 weeks) groups. Outcomes included behavioral and emotional problems, cognitive function, structural magnetic resonance imaging (MRI), diffusion tensor imaging (DTI), and resting-state functional MRI. Longitudinal mixed-effects models were used as the primary analyses, with Type III tests evaluating group, timepoint, and group-by-timepoint effects. False discovery rate correction was applied within predefined outcome families.

**Results:**

The longitudinal models showed no broad LP–FT differences or group-by-timepoint interactions in behavioral and emotional problems, most cognitive outcomes, cortical thickness, cortical surface area, or resting state functional MRI within-network connectivity. Structural MRI showed group effects in six regional brain volumes, without significant timepoint or group-by-timepoint effects. DTI showed the most prominent findings: significant group effects were observed in selected white matter tract, cortical and subcortical region of interest fractional anisotropy or mean diffusivity metrics, and significant group-by-timepoint interactions were observed in selected DTI metrics.

**Discussion:**

These findings suggest that late preterm birth is associated with subtle and regionally specific neuroimaging differences during early adolescence, particularly in DTI metrics.

## 1 Introduction

A preterm infant is defined as one born before 37 weeks of gestation. The World Health Organization classifies preterm birth into extremely preterm (<28 weeks), very preterm (28 to <32 weeks), and moderate-to-late preterm (32 to <37 weeks) (1). According to the National Institute of Child Health and Human Development, late preterm (LP) infants are defined as those born between 34 and 36 weeks of gestation (2), accounting for 3%–6% of all births worldwide (3).

In the short term, LP infants have a higher risk of various complications, including respiratory distress syndrome, intraventricular hemorrhage, hypothermia, and hypoglycemia, as well as higher post-discharge readmission rates, than full-term (FT) infants (4, 5). Several studies have examined the long-term outcomes of LP children. LP children are more likely to experience difficulties in communication and attention during infancy and adolescence (6–8) and have a higher risk of autism spectrum disorder or attention deficit hyperactivity disorder than FT children (9, 10). Cognitive and language delays may also impact their academic performance and social interactions (11–13). Meanwhile, some studies have reported no significant differences in cognitive function or neurodevelopmental disorders between LP and FT children (14–20). Therefore, a consensus regarding the long-term outcomes of LP children has not been reached.

After 34 weeks of gestation, the brain undergoes rapid development, with white matter volume increasing approximately five-fold by 40 weeks (21). This period is characterized by synaptogenesis, dendritic growth, increased brain surface area, and gyrification (22). Exposure to the extrauterine environment at this time may disrupt these processes (23), leading to subtle alterations in brain maturation in LP children compared with FT children, though generally less pronounced than in more premature groups (24). Even in the absence of apparent complications early in life, LP children remain at risk of neurodevelopmental disorders, owing to maturation disorders of the cerebral cortex (25).

Previous magnetic resonance imaging (MRI) studies have provided further insights into brain development in LP children. Structural MRI (sMRI) studies have reported smaller brain and gray matter volumes with delayed cortical gyrification in LP children at term-equivalent age (TEA) (26, 27), and brain volumes at TEA have been associated with neurodevelopmental outcomes at 2 years (28). Longitudinal findings suggest that lower gestational age is associated with reduced cortical volume in adolescence, influencing cognitive and educational performance (29), and altered gray matter volume has also been related to anxiety symptoms at school age (30). Diffusion tensor imaging (DTI) studies have revealed altered diffusion indices in premature infants aged 32–36 weeks (31), with regional white matter microstructural differences associated with neurodevelopmental outcomes (32). Functional MRI (fMRI) studies also reveal altered network development: LP infants demonstrate changes in dynamic functional connectivity with brain maturity (33), and resting-state fMRI (rs-fMRI) has shown disrupted networks involving the prefrontal and anterior cingulate cortices in LP populations (34).

However, these previous studies were based on relatively small samples or combined moderate preterm and LP children aged 32–33 weeks, thus leaving the long-term neurodevelopmental trajectory of LP children still unclear.

Therefore, in this study, we aimed to examine behavioral, cognitive, and multimodal neuroimaging outcomes in children born late preterm during early adolescence using data from the large-scale, United States–based Adolescent Brain Cognitive Development (ABCD) Study. We compared LP children with FT children and used longitudinal mixed-effects models incorporating group, timepoint, and group-by-timepoint interaction terms as the primary analytic framework. These models were used to evaluate overall LP–FT differences, developmental changes from baseline to the two-year follow-up, and whether two-year changes differed between the LP and FT children across behavioral and emotional problems, cognitive function, sMRI measures, DTI metrics, and resting-state functional connectivity. Descriptive baseline analyses were retained as supplementary timepoint-specific analyses to characterize LP–FT differences at 9–11 years of age.

## 2 Materials and methods

### 2.1 Participants

We utilized data from the ABCD Study—a large-scale, longitudinal cohort study conducted in the United States (35). As data from the ABCD study are stored in the NIMH Data Archive, all methods were performed in accordance with the NIMH Data Archive Data Use Certification Agreement. Informed consent for participants in the ABCD study was obtained from parents for their own participation and that of their minor child, and the minor also provided his or her assent. We analyzed data from the ABCD 3.0 release, which included 11,878 adolescents aged 9–11 years [mean age, 118.98 months (min, 107; max, 133)] at baseline recruited from 21 data collection sites. All participants provided written informed consent. All procedures adhered to the principles outlined in the Declaration of Helsinki. The Research Ethics Committee of the University of Fukui approved the data analysis (approval no. Fu-20210067).

We analyzed both baseline and 2-year follow-up data. Gestational age at birth was obtained from retrospective parent/caregiver report, and participants were classified as LP (34–36 weeks) or FT (≥ 40 weeks). Specifically, parents were asked, “Was the child born prematurely?” and “About how many weeks premature was the child at birth?” (29). Supplementary material outlines the inclusion and exclusion criteria (see Supplementary Figures S1, S2, and S3 illustrate the study flowchart). Data cleaning (R package “dplyr”) and statistical analyses were conducted using R (version 4.3.0; The R Foundation for Statistical Computing, Vienna, Austria).

### 2.2 Demographic variables and covariates

We collected demographic and clinical data for various covariates, including age at assessment, sex, handedness, race/ethnicity, annual household income, parental educational level, pubertal status, body mass index (BMI), and maternal age at birth. Available perinatal characteristics, including birth weight, mode of delivery, maternal complications, days in incubator, and birth distress–related neonatal indicators (i.e., “blue at birth [pale],” “slow heart beat [bradycardia],” and “did not breathe at first [apnea]”), were extracted to further characterize the cohort (Table 1). These perinatal variables were not included in the primary adjusted models because several of them may lie on the causal pathway between gestational age and later neurodevelopmental outcomes.

**Table 1:** Demographic characteristics of the ABCD participants at baseline and 2-year follow-up.

	Baseline			2-year follow-up		
Behavioral/emotional, cognitive, and sMRI analysis sample	LP, *n* = 1,093	FT, *n* = 8,984	*p*-value	LP, *n* = 533	FT, *n* = 4,223	*p*-value
Age^a^ [months]	120.1 (7.22)	118.7 (7.53)	<0.001^c^	144.5 (7.66)	142.8 (7.63)	<0.001^c^
Sex^b^ [male/female]	571/522	4,653/4,331	0.804^d^	292/241	2,260/1,963	0.612^d^
BMI^a^	18.4 (3.82)	18.8 (3.98)	0.005^c^	20.5 (4.72)	20.5 (4.65)	0.972^c^
Annual household income^b^						
<$50,000	220	2,452	<0.001^d^	－	－	
$50,000–74,999	155	1,126	<0.001^d^	－	－	
$75,000–99,999	157	1,216	<0.001^d^	－	－	
$100,000–199,999	378	2,485	<0.001^d^	－	－	
>$200,000	121	943	<0.001^d^	－	－	
Parental education^a^ [years]	15.5 (2.24)	15.2 (2.56)	<0.001^c^	－	－	
Race^b^						
White	664	4,682	<0.001^d^	－	－	
Black	142	1,300	<0.001^d^	－	－	
Hispanic	166	1,885	<0.001^d^	－	－	
Asian	10	188	<0.001^d^	－	－	
Puberty score^a^	1.61 (0.39)	1.64 (0.41)	0.130^c^	2.06 (0.62)	2.08 (0.62)	0.479^c^
Maternal age at birth^a^ [years]	30.2 (6.14)	29.2 (6.27)	<0.001^c^	－	－	
History of cesarean section^b^	654	2,963	<0.001^d^	－	－	
Pregnancy related high blood pressure^b^	198	652	<0.001^d^	－	－	
Pregnancy related diabetes^b^	102	541	<0.001^d^	－	－	
Birth body weight^a^ [kg]	2.43 (0.48)	3.36 (0.54)	<0.001^c^	－	－	
Complications at birth, pale^b^	51	241	<0.001^d^	－	－	
Complications at birth, bradycardia^b^	28	202	0.53^d^	－	－	
Complications at birth, temporary apnea^b^	75	347	<0.001^d^	－	－	
Duration of incubator care^a^ [days]	2.43 (3.27)	0.2 (0.96)	<0.001^c^	－	－	

DTI analysis sample	LP, *n* = 997	FT, *n* = 8,193	*p*-value	LP, *n* = 462	FT, *n* = 3,662	*p*-value
Age^a^ [months]	120.1 (7.23)	118.8 (7.54)	<0.001^c^	144.7 (7.74)	142.9 (7.63)	<0.001^c^
Sex^b^ [male/female]	506/491	4,228/3,965	0.635	255/207	1,957/1,705	0.507^d^
BMI^a^	18.3 (3.69)	18.7 (3.98)	0.003^c^	20.3 (4.65)	20.4 (4.60)	0.540^c^
Annual household income^b^						
<$50,000	192	2,179	<0.001^d^	－	－	
$50,000–74,999	143	1,005	<0.001^d^	－	－	
$75,000–99,999	140	1,121	<0.001^d^	－	－	
$100,000–199,999	349	2,322	<0.001^d^	－	－	
>$200,000	116	876	<0.001^d^	－	－	
Parental education^a^ [years]	15.6 (2.25)	15.3 (2.54)	0.001^c^	－	－	
Race^b^						
White	613	4,344	<0.001^d^	－	－	
Black	126	1,150	<0.001^d^	－	－	
Hispanic	151	1,703	<0.001^d^	－	－	
Asian	8	161	<0.001^d^	－	－	
Puberty score^a^	1.61 (0.39)	1.63 (0.41)	0.364^c^	2.05 (0.61)	2.08 (0.62)	0.364^c^
Maternal age at birth^a^ [years]	30.3 (6.00)	29.3 (6.25)	<0.001^c^	－	－	
History of cesarean section^b^	588	2,679	<0.001^d^	－	－	
Pregnancy related high blood pressure^b^	176	573	<0.001^d^	－	－	
Pregnancy related diabetes^b^	92	493	<0.001^d^	－	－	
Birth body weight^a^ [kg]	2.43 (0.48)	3.35 (0.53)	<0.001^c^	－	－	
Complications at birth, pale^b^	51	241	<0.001^d^	－	－	
Complications at birth, bradycardia^b^	23	180	0.853^d^	－	－	
Complications at birth, temporary apnea^b^	68	303	<0.001^d^	－	－	
Duration of incubator care^a^ [days]	2.24 (3.27)	0.2 (0.95)	<0.001^c^	－	－	

rs-fMRI analysis sample	LP, *n* = 523	FT, *n* = 4,547	*p*-value	LP, *n* = 212	FT, *n* = 1,813	*p*-value
Age^a^ [months]	121.1 (7.25)	119.6 (7.55)	<0.001^c^	145.5 (7.70)	143.8 (7.76)	0.002^c^
Sex^b^ [male/female]	236/287	2,183/2,364	0.230^d^	100/112	860/953	0.999^d^
BMI^a^	17.9 (3.32)	18.3 (3.60)	0.011^c^	19.9 (4.19)	19.7 (4.11)	0.747^c^
Annual household income^b^						
<$50,000	81	1,093	<0.001^d^	－	－	
$50,000–74,999	75	558	<0.001^d^	－	－	
$75,000–99,999	71	657	<0.001^d^	－	－	
$100,000–199,999	193	1,343	<0.001^d^	－	－	
>$200,000	72	549	<0.001^d^	－	－	
Parental education^a^ [years]	15.7 (2.28)	15.5 (2.48)	0.100^c^	－	－	
Race^b^						
White	345	2,588	<0.001^d^	－	－	
Black	50	519	<0.001^d^	－	－	
Hispanic	74	863	<0.001^d^	－	－	
Asian	4	88	<0.001^d^	－	－	
Puberty score^a^	1.61 (0.39)	1.64 (0.41)	0.820^c^	2.13 (0.63)	2.11 (0.64)	0.677^c^
Maternal age at birth^a^ [years]	30.3 (6.00)	29.3 (6.25)	0.005^c^	－	－	
History of cesarean section^b^	317	1,459	<0.001^d^	－	－	
Pregnancy related high blood pressure^b^	89	300	<0.001^d^	－	－	
Pregnancy related diabetes^b^	47	262	0.0048^d^	－	－	
Birth body weight^a^ [kg]	2.42 (0.47)	3.36 (0.53)	<0.001^c^	－	－	
Complications at birth, pale^b^	31	108	<0.001^d^	－	－	
Complications at birth, bradycardia^b^	14	94	0.412^d^	－	－	
Complications at birth, temporary apnea^b^	45	157	<0.001^d^	－	－	
Duration of incubator care^a^ [days]	2.4 (3.29)	0.18 (0.92)	<0.001^c^	－	－	

Demographic characteristics included in all analyses: behavioral and emotional problems, cognitive function, brain structure, DTI, and rs-fMRI data.

a Data are presented as the mean ± standard deviation for each measure.

b Data are presented as the number of participants (n).

c Statistical comparisons were performed using the Mann–Whitney U test.

d Categorical variables were analyzed using the chi-square test.

sMRI, structure MRI; BMI, body mass index; DTI, diffusion tensor imaging; FT, full-term; LP, late preterm; rs-fMRI, resting-state functional magnetic resonance imaging; ABCD, Adolescent Brain Cognitive Development.

Categorical variables, which were dummy-coded, included sex, handedness, and race/ethnicity (White, Black, Hispanic, and Asian). Continuous variables included age, parental educational level, puberty score, BMI, and maternal age at birth. Based on previous studies (36, 37), annual household income was considered a five-level categorical variable. Parental educational level was categorized as follows: 12th grade or less (12 years), college and associate degrees (14 years), bachelor's degree (16 years), master's degree (18 years), and professional/doctoral degree (20 years). Puberty status was assessed using a puberty score (38), which was completed by both a parent/guardian and the child, with their average being used as the final value. Children whose mothers reported tobacco, alcohol, or cannabis use were excluded from the analyses.

The same covariate framework was used for the primary longitudinal mixed-effects models and descriptive timepoint-specific analyses. Time-invariant covariates included sex, handedness, race/ethnicity, parental education, household income, and maternal age at birth. Time-varying covariates, including age at assessment, BMI, pubertal score, and intracranial volume (ICV), were entered using values from the corresponding timepoint in the longitudinal models. For descriptive baseline analyses, baseline values were used. ICV was included in all neuroimaging models to account for overall brain size.

### 2.3 Behavioral and emotional problems

Behavioral and emotional problems were assessed using the Child Behavior Checklist (39). A T-score >65 on the fifth edition of the Diagnostic and Statistical Manual of Mental Disorders-oriented scales for Depression, Anxiety disorders, Somatic problems, Attention Deficit Hyperactivity Disorder, Oppositional Defiant Disorder, and Conduct Disorder indicated clinically significant symptomatology (see Supplementary Material for the details).

### 2.4 Cognitive function

Cognitive function—executive function, processing speed, episodic memory, and language—was assessed using the National Institutes of Health Toolbox (40–42). Cognitive tests included the flanker inhibitory control and attention task and the dimensional change card sort (for executive function), pattern-comparison-processing speed task (for processing speed), picture sequence memory task (for episodic memory), list sorting working memory task (for working memory), and picture vocabulary and oral reading recognition tasks (for language functioning; see Supplementary material for task details) (40–42). Age-corrected scores were used for all cognitive measures. Moreover, the Rey Auditory Verbal Learning Test (RAVLT) was employed to assess delayed recall as part of memory assessment (29). The RAVLT measures included short-delay and long-delay memory scores. These nine cognitive outcomes were analyzed together as the cognitive function domain.

### 2.5 Image acquisition and processing

Neuroimaging data were acquired at 21 different sites and processed by the ABCD team. The standard scan session of the ABCD Study included structural MRI (T1w and T2), DTI, and rs-fMRI series (43). Each atlas used for image analysis was selected based on its suitability for the respective imaging modality, ensuring optimized anatomical and functional parcellation across analyses.

### 2.6 Structural MRI data

High-resolution T1-weighted three-dimensional structural images (1 mm isotropic) were obtained using three 3 T MRI scanners (Siemens, General Electric 750, and Philips) with acquisition parameters as previously described (43).

Brain structural data were preprocessed using the standard morphometric pipeline (i.e., skull-stripping and white matter segmentation) in FreeSurfer (version 5.3.0) (44). We extracted the brain volume, cortical thickness, and surface area of 34 cortical brain regions in each hemisphere using the Desikan–Killiany atlas. Additionally, we extracted the volumes of seven subcortical regions in each hemisphere labeled by atlas-based segmentation (45, 46).

### 2.7 DTI data

DTI acquisition (1.7 mm isotropic) was performed using multiband echo planar imaging, with a slice acceleration factor of 3. The protocol included 96 diffusion directions and seven b=0 frames, distributed across four different b-values (6 directions with b=500 s/mm^2^; 15 directions with b=1,000 s/mm^2^, 15 directions with b=2,000 s/mm^2^, and 60 directions with b=3,000 s/mm^2^) (44).

We extracted 35 major white matter tracts using the AtlasTract (46). These included three non-lateralized/callosal tracts—the forceps major, forceps minor, and corpus callosum—and 16 bilateral tracts—the fornix, cingulate cingulum, parahippocampal cingulum, corticospinal/pyramidal tract, anterior thalamic radiation, uncinate fasciculus, inferior longitudinal fasciculus, inferior fronto-occipital fasciculus, superior longitudinal fasciculus, temporal superior longitudinal fasciculus, parietal superior longitudinal fasciculus, superior corticostriate, superior corticostriate-frontal cortex, superior corticostriate-parietal cortex, striatal inferior frontal cortex, and inferior frontal superior frontal cortex. Additionally, we extracted 34 cortical regions of interest (ROIs) per hemisphere (68 in total), based on the Desikan–Killiany atlas classification (45), and seven bilateral subcortical ROIs (14 in total), based on Automatic Subcortical Segmentation. White matter tract parameters included fractional anisotropy (FA) and mean diffusivity (MD) (47), which reflect the directionality of tissue properties and diffusion, respectively. We excluded data that did not meet the quality control criteria for DTI imaging (44).

### 2.8 Resting-state functional magnetic resonance data

Rs-fMRI data processing was performed by the ABCD Data Analysis and Informatics Core using the standardized ABCD pipeline. Rs-fMRI acquisition (2.4 mm isotropic, TR = 800 ms) was performed using multiband echo planar imaging with a slice acceleration factor of 6 and 20 min of resting state activity (44). We excluded data that did not meet the quality control criteria for rs-fMRI.

We examined within-network connectivity based on the Gordon parcellation scheme (48) for the following 12 predefined resting-state networks: auditory network, cingulo-opercular network, cingulo-parietal network, default mode network, dorsal attention network, fronto-parietal network, retrosplenial temporal network, sensorimotor hand network, sensorimotor mouth network, salience network, ventral attention network, and visual network.

### 2.9 Statistical analysis

All statistical analyses were performed using R (version 4.3.0; The R Foundation for Statistical Computing, Vienna, Austria). Outliers were winsorized at ±3 standard deviations from the mean using the ‘‘DeseTools’' package. Linear mixed-effects models were fitted using the ‘‘lmerTest’' package. Type III tests of fixed effects were performed using Satterthwaite's approximation for degrees of freedom. Sum-to-zero contrasts were used for categorical predictors when performing Type III tests.

#### 2.9.1 Primary longitudinal mixed-effects analyses

The primary analyses comprised longitudinal mixed-effects modeling using data from both baseline and at two-year follow-up. For each outcome, the model included group, timepoint, and the group-by-timepoint interaction as fixed effects. Type III tests of fixed effects were used to evaluate the main effect of group, main effect of timepoint, and group-by-timepoint interaction. The group effect tested overall LP–FT differences within the longitudinal model, timepoint effect tested baseline-to-follow-up changes, and group-by-timepoint interaction tested whether longitudinal changes differed between the LP and FT children. Sum-to-zero contrasts were used for categorical predictors when performing Type III tests.

Random intercepts for participant ID, family ID, and data collection site were included in the longitudinal models (49, 50). Participant ID accounted for repeated measurements within individuals, family ID accounted for familial clustering, and data collection site accounted for site-level clustering. Twin/triplet status and MRI scanner information were included as fixed-effect covariates to avoid redundancy with family ID and data collection site, respectively.

These longitudinal models were applied across all the examined outcomes, including behavioral and emotional problems (6 outcomes), cognitive function (9 outcomes), regional brain volumes (82 regions), cortical thickness (68 regions), cortical surface area (68 regions), DTI white matter tract FA and MD (35 tracts each), DTI cortical ROI FA and MD (68 ROIs each), DTI subcortical ROI FA and MD (14 ROIs each), and rs-fMRI within-network connectivity (12 networks). The same covariate framework was applied across outcome domains. Time-varying covariates, including age at assessment, BMI, pubertal score, and ICV, were entered using timepoint-specific values. ICV was included as a covariate in all neuroimaging models.

#### 2.9.2 Descriptive timepoint-specific analyses

In addition to the primary longitudinal analyses, descriptive timepoint-specific analyses were conducted to characterize LP–FT differences at baseline and to facilitate comparison with previous cross-sectional reports. These baseline analyses were not used as the primary test for longitudinal change and are reported in the Supplementary Materials.

In the baseline models, the outcome measure was entered as the dependent variable and group as the primary independent variable. Family ID and data collection site were included as random intercepts. Twin/triplet status was included as a fixed-effect covariate, and MRI scanner information was included as a fixed-effect covariate for neuroimaging models. The same covariate framework described above was employed, with baseline values used for time-varying covariates.

#### 2.9.3 Post hoc simple-effects analyses

Post hoc simple-effects analyses were conducted to characterize the direction of interactions for outcome measures showing significant group-by-timepoint interactions after false discovery rate (FDR) correction. These analyses examined LP–FT differences at each timepoint and follow-up-to-baseline changes within each group using the same longitudinal mixed-effects models. The *post hoc* analyses were considered descriptive and were used only to aid interpretation of significant interaction terms.

#### 2.9.4 Multiple comparison correction

To account for multiple comparisons, *p*-values were adjusted using the Benjamini–Hochberg FDR procedure. For the primary longitudinal mixed-effects analyses, FDR correction was applied within each predefined outcome family and separately for each fixed effect tested in the Type III models, namely the group main effect, timepoint main effect, and group-by-timepoint interaction. For descriptive timepoint-specific analyses and *post hoc* simple-effects analyses, FDR correction was applied within the corresponding outcome family or comparison set. Statistical significance was defined as an FDR-adjusted *p*-value < 0.05.

#### 2.9.5 Attrition analysis

To evaluate potential attrition bias at the 2-year follow-up, we compared baseline sMRI (brain volume) and DTI metrics between the completers and non-completers. Completers were defined as participants with complete data for the corresponding imaging domain at both baseline and two-year follow-up. Non-completers were defined as participants with complete baseline data but incomplete or unavailable follow-up data for that domain. For each imaging domain, we first calculated the overall retention rate and compared the retention rates between the LP and FT children using *χ*^2^ tests. We then compared baseline demographic, socioeconomic, behavioral, cognitive, pubertal, data collection site, MRI scanner, and neuroimaging characteristics between the completers and non-completers. Standardized mean differences (SMDs) were calculated to quantify baseline imbalance, with SMD values ≥ 0.1 considered indicative of potential imbalance. For imaging domains with significant baseline group differences, the corresponding baseline imaging measures were included in the completer vs. non-completer comparisons to evaluate whether the reported baseline findings were affected by differential follow-up data availability. In addition, SMDs were examined across all baseline DTI tract/ROI measures within each DTI domain. Finally, we fitted logistic regression models to examine whether availability of follow-up data was associated with LP/FT group status after adjustment for key baseline covariates. These attrition analyses were used to assess potential bias in the follow-up and longitudinal findings (51, 52).

## 3 Results

The primary results are based on longitudinal mixed-effects models using Type III tests for fixed effects. These models evaluated the main effects of group and timepoint and the group-by-timepoint interaction. Descriptive baseline analyses were retained in the Supplementary Materials to characterize LP–FT differences at 9–11 years of age but were not used as the primary test of longitudinal effects (see Supplementary Tables S1). Complete Type III test results are provided in Supplementary Tables S2–S8.

### 3.1 Behavioral and emotional problems and cognitive function

In the longitudinal mixed-effects models for behavioral and emotional problems, no significant group effects or group-by-timepoint interactions were observed after FDR correction. A significant timepoint effect was observed only for somatic problems, suggesting an overall change from baseline to the two-year follow-up across groups (see Supplementary Tables S2).

For cognitive function, flanker showed a significant group effect within the longitudinal model, whereas no cognitive outcome showed a significant group-by-timepoint interaction. Several cognitive measures, including picture vocabulary, flanker, pattern comparison, picture sequence, reading, short memory, and long memory, showed significant timepoint effects, indicating overall changes from baseline to follow-up (see Supplementary Tables S3).

### 3.2 Brain volume, cortical thickness, and cortical surface area

Type III tests from the longitudinal mixed-effects models showed significant group effects in six regional brain volume measures, including the left postcentral, right fusiform, right middle temporal, right pars orbitalis, right supramarginal, and right insular regions. No significant timepoint effects or group-by-timepoint interactions were observed for regional brain volume measures after FDR correction (see Table 2 and Supplementary Table S4). For cortical thickness and cortical surface area, no significant group, timepoint, or group-by-timepoint effects were observed after FDR correction (see Supplementary Tables S5 and S6).

**Table 2:** Longitudinal mixed-effects model results for brain volume measures showing significant group main effects or group-by-timepoint interactions.

Region	Group effect F (FDR-p)	Timepoint effect F (FDR-p)	Group×Timepoint effect F (FDR-p)
Lt. Post central	**8.54 (0.048)**	0.49 (0.839)	0.04 (0.930)
Rt. Fusiform	**9.24 (0.048)**	0.01 (0.965)	2.17 (0.919)
Rt. Middle temporal	**13.9 (0.008)**	0.14 (0.942)	3.15 (0.919
Rt. Pars orbitalis	**16.61 (0.004)**	0.39 (0.894)	0.85 (0.919)
Rt. Supra marginal	**11.25 (0.022)**	2.69 (0.489)	0.30 (0.919)
Rt. Insula	**8.85 (0.048)**	0.79 (0.767)	0.05 (0.930)

Values are F statistics with FDR-adjusted *p*-values in parentheses. Type III tests of fixed effects were obtained from longitudinal mixed-effects models. The group effect tests overall LP–FT differences within the longitudinal model, timepoint effect tests baseline-to-follow-up changes, and group-by-timepoint effect tests whether longitudinal changes differed between the LP and FT children. This table includes brain volume regions showing significant group effects after FDR correction; complete Type III results for all brain volume outcomes are provided in Supplementary Tables S4. Models included group, timepoint, and group-by-timepoint as fixed effects, with participant ID, family ID, and data collection site as random intercepts. Twin/triplet status and MRI scanner information were included as fixed-effect covariates. For neuroimaging outcomes, intracranial volume was included as a timepoint-specific covariate, with baseline ICV used for baseline observations and follow-up ICV used for follow-up observations. FDR correction was applied within each predefined outcome family and effect type. FDR-adjusted *p* < 0.05 was considered statistically significant. Bold values indicate these significant results. LP, late preterm; FT, full term; FDR, false discovery rate; ICV, intracranial volume; Lt., left; Rt., right.

### 3.3 DTI

#### 3.3.1 White matter tracts

Type III tests from the longitudinal mixed-effects models showed significant effects in selected FA and MD metrics (see Table 3).

**Table 3:** Longitudinal mixed-effects model results for white matter tracts FA and MD metrics showing significant group main effects or group-by-timepoint interactions.

DTI metrics	White matter tracts	Group effect F (FDR-p)	Timepoint effect F (FDR-p)	Group×timepoint F (FDR-p)
FA	Lt. Fornix	0.00 (0.993)	**12.81 (0.003)**	**6.86 (0.046)**
	Lt. Anterior thalamic radiation	0.21 (0.911)	**5.74 (0.041)**	**17.48 (0.001)**
	Lt. Uncinate	0.50 (0.799)	0.65 (0.624)	**9.70 (0.022)**
	Lt. Inferior longitudinal fasciculus	6.62 (0.164)	0.38 (0.695)	**6.50 (0.046)**
	Lt. Inferior fronto-occipital fasciculus	0.07 (0.973)	1.71 (0.371)	**6.48 (0.046)**
	Lt. Striatal inferior frontal cortex	0.01 (0.993)	0.17 (0.771)	**13.95 (0.003)**
	Rt. Fornix	2.42 (0.467)	**7.19 (0.029)**	**7.13 (0.046)**
	Rt. Inferior longitudinal fasciculus	**10.9 (0.034)**	0.25 (0.721)	2.45 (0.293)
	Rt. Superior corticostriate	5.23 (0.195)	1.36 (0.427)	**6.47 (0.046)**
	Rt. Superior corticostriate-parietal cortex	6.03 (0.164)	0.01 (0.905)	**6.33 (0.046)**
MD	Lt. Cingulate cingulum	0.33 (0.659)	0.11 (0.766)	**7.91 (0.039)**
	Lt. Inferior longitudinal fasciculus	**14.53 (0.005)**	**7.70 (0.007)**	0.03 (0.967)
	Lt. Striatal inferior frontal cortex	6.91 (0.089)	2.03 (0.169)	**7.28 (0.041)**
	Rt. Inferior longitudinal fasciculus	5.38 (0.101)	**18.45 (<0.001)**	**7.85 (0.039)**
	Rt. Inferior fronto-occipital fasciculus	1.91 (0.293)	**8.44 (0.005)**	**8.75 (0.039)**
	Rt. Superior corticostriate	1.18 (0.374)	**37.42 (<0.001)**	**7.71 (0.039)**
	Rt. Superior corticostriate-parietal cortex	1.19 (0.374)	**39.22 (<0.001)**	**8.17 (0.039)**

Values are F statistics with FDR-adjusted *p*-values in parentheses. Type III tests of fixed effects were obtained from longitudinal mixed-effects models. The group effect tests overall LP–FT differences within the longitudinal model, timepoint effect tests baseline-to-follow-up changes, and group-by-timepoint effect tests whether longitudinal changes differed between the LP and FT children. This table includes measures showing significant group effects or significant group x timepoint effects after FDR correction; complete Type III results for all white matter tracts FA and MD measures are provided in Supplementary Tables S7–S1 and S7-2. Models included group, timepoint, and group-by-timepoint as fixed effects, with participant ID, family ID, and data collection site as random intercepts. Twin/triplet status and MRI scanner information were included as fixed-effect covariates. For neuroimaging outcomes, intracranial volume was included as a timepoint-specific covariate, with baseline ICV used for baseline observations and follow-up ICV used for follow-up observations. FDR correction was applied within each predefined outcome family and effect type. FDR-adjusted *p* < 0.05 was considered statistically significant. Bold values indicate these significant results. LP, late preterm; FT, full term; FDR, false discovery rate; FA, fractional anisotropy; MD, mean diffusivity; ICV, intracranial volume; Lt., left; Rt., right.

For white matter tracts FA, a significant group effect was observed in the right inferior longitudinal fasciculus (ILF), indicating an overall LP–FT difference within the longitudinal model. Significant group-by-timepoint interactions were observed in nine white matter tracts FA measures: left fornix, left anterior thalamic radiation, left uncinate fasciculus, left ILF, left inferior fronto-occipital fasciculus, left striatal inferior frontal cortex, right fornix, right superior corticostriate, and right superior corticostriate-parietal cortex. Significant timepoint effects were observed in the left fornix, left anterior thalamic radiation, and right fornix.

For white matter tracts MD, a significant group effect was observed in the left inferior longitudinal fasciculus, and significant group-by-timepoint interactions were observed in six tracts: left cingulate cingulum, left striatal inferior frontal cortex, right ILF, right inferior fronto-occipital fasciculus, right superior corticostriate, and right superior corticostriate-parietal cortex. Significant timepoint effects were observed in the left ILF, right ILF, right inferior fronto-occipital fasciculus, right superior corticostriate, and right superior corticostriate-parietal cortex.

Post hoc analyses for white matter tract measures with significant group-by-timepoint interactions showed that several white matter tracts FA interactions were characterized by significant follow-up-to-baseline decreases in FT children, whereas LP children generally showed smaller or non-significant changes. Some white matter tracts FA measures showed no significant LP–FT differences at baseline but showed significant differences at follow-up, including the left ILF and right superior corticostriate-related tracts. For white matter tracts MD, several right-sided tracts showed significant LP–FT differences at baseline that were not observed at follow-up, with significant decreases over time in FT children and in selected tracts in LP children (see Supplementary Table S9).

#### 3.3.2 Cortical ROIs

For cortical ROI FA metrics, Type III tests from the longitudinal mixed-effects models showed significant group effects in 13 ROIs: bilateral lingual, bilateral middle temporal, bilateral pericalcarine, left precuneus, left rostral anterior cingulate, bilateral superior temporal, left supramarginal, left transverse temporal, and right rostral middle frontal ROIs. Significant timepoint effects were observed in four cortical ROI FA metrics, including the left pericalcarine, left supramarginal, left transverse temporal, and right pericalcarine ROIs. A significant group-by-timepoint interaction was observed only in the left rostral anterior cingulate.

For cortical ROI MD metrics, no significant group effects were observed after FDR correction. Significant timepoint effects and group-by-timepoint interactions were observed in eight right-hemisphere cortical ROIs: middle temporal, pars opercularis, pars triangularis, postcentral, precentral, superior temporal, supramarginal, and insula (see Table 4).

**Table 4:** Longitudinal mixed-effects model results for cortical FA and MD metrics showing significant group main effects or group-by-timepoint interactions.

DTI metrics	Cortical ROIs	Group effect F (FDR-p)	Timepoint effect F (FDR-p)	Group×timepoint F (FDR-p)
FA	Lt. Lingual	**11.49 (0.007)**	4.46 (0.068)	1.78 (0.487)
	Lt. Middle temporal	**7.57 (0.034)**	1.10 (0.416)	2.35 (0.426)
	Lt. Peri calcarine	**13.58 (0.004)**	**17.28 (<0.001)**	0.04 (0.948)
	Lt. Precuneus	**9.05 (0.018)**	1.77 (0.278)	3.45 (0.287)
	Lt. Rostral anterior cingulate	**18.78 (0.001)**	2.07 (0.244)	**18.68 (0.001)**
	Lt. Superior temporal	**12.11 (0.007)**	0.12 (0.840)	4.84 (0.201)
	Lt. Supra marginal	**7.83 (0.032)**	**6.41 (0.029)**	0.006 (0.983)
	Lt. Transverse temporal	**9.43 (0.016)**	**11.49 (0.003)**	0.12 (0.937)
	Rt. Lingual	**13.47 (0.004)**	1.87 (0.265)	0.76 (0.743)
	Rt. Middle temporal	**14.10 (0.004)**	0.48 (0.628)	1.73 (0.487)
	Rt. Peri calcarine	**11.54 (0.007)**	**5.80 (0.038)**	0.36 (0.909)
	Rt. Rostral middle frontal	**9.53 (0.016)**	4.23 (0.074)	2.57 (0.412)
	Rt. Superior temporal	**7.14 (0.039)**	1.49 (0.321)	3.93 (0.248)
MD	Rt. Middle temporal	1.19 (0.612)	**16.70 (<0.001)**	**8.44 (0.031)**
	Rt. Pars opercularis	0.11 (0.895)	**23.60 (<0.001)**	**15.23 (0.007)**
	Rt. Pars triangularis	0.02 (0.971)	**11.37 (0.001)**	**11.32 (0.018)**
	Rt. Post central	0.36 (0.788)	**37.65 (<0.001)**	**9.66 (0.027)**
	Rt. Precentral	0.06 (0.965)	**27.00 (<0.001)**	**12.30 (0.016)**
	Rt. Superior temporal	2.14 (0.480)	**22.79 (<0.001)**	**8.96 (0.027)**
	Rt. Supra marginal	0.49 (0.764)	**57.28 (<0.001)**	**9.07 (0.027)**
	Rt. Insula	0.03 (0.971)	**5.86 (0.020)**	**9.49 (0.027)**

Values are F statistics with FDR-adjusted *p*-values in parentheses. Type III tests of fixed effects were obtained from longitudinal mixed-effects models. The group effect tests overall LP–FT differences within the longitudinal model, timepoint effect tests baseline-to-follow-up changes, and group-by-timepoint effect tests whether longitudinal changes differed between the LP and FT children. This table includes measures showing significant group effects or significant group-by-timepoint effects after FDR correction; complete type III results for all cortical ROIs FA and MD measures are provided in Supplementary Tables S7–S3 and S7–S4. Models included group, timepoint, and group-by-timepoint as fixed effects, with participant ID, family ID, and data collection site as random intercepts. Twin/triplet status and MRI scanner information were included as fixed-effect covariates. For neuroimaging outcomes, intracranial volume was included as a timepoint-specific covariate, with baseline ICV used for baseline observations and follow-up ICV used for follow-up observations. FDR correction was applied within each predefined outcome family and effect type. FDR-adjusted *p* < 0.05 was considered statistically significant. Bold values indicate these significant results. LP, late preterm; FT, full term; ROIs, region of interest; FDR, false discovery rate; FA, fractional anisotropy; MD, mean diffusivity; ICV, intracranial volume; Lt., left; Rt., right.

Cortical ROI metrics showing significant group-by-timepoint interactions underwent *post hoc* simple-effects analyses. For FA, the left rostral anterior cingulate showed no significant LP–FT difference at baseline but demonstrated a significant LP–FT difference at follow-up, with a significant follow-up-to-baseline decrease in FT children but not in LP children. For MD, FT children showed significant follow-up-to-baseline decreases in ROIs with significant interactions, whereas LP children showed significant decreases only in selected regions (see Supplementary Table S9).

#### 3.3.3 Subcortical ROIs

For subcortical ROI FA metrics, Type III tests from the longitudinal mixed-effects models showed significant group effects in eight ROIs: left caudate, bilateral putamen, bilateral pallidum, bilateral hippocampus, and right amygdala. Significant timepoint effects were observed in eight subcortical FA metrics, including left caudate, left putamen, left pallidum, left amygdala, right thalamus, right putamen, right pallidum, and right hippocampus. Significant group-by-timepoint interactions were observed in six subcortical FA metrics: left putamen, left pallidum, left hippocampus, left amygdala, right thalamus, and right pallidum.

For subcortical ROI MD metrics, a significant group effect was observed only in the right pallidum. Significant timepoint effects and group-by-timepoint interactions were observed in bilateral hippocampal MD. No significant timepoint effect or group-by-timepoint interaction was observed for the right pallidum MD (see Table 5).

**Table 5:** Longitudinal mixed-effects model results for subcortical FA and MD metrics showing significant group main effects or group-by-timepoint interactions.

DTI metrics	Subcortical ROIs	Group effect F (FDR-p)	Timepoint effect F (FDR-p)	Group×timepoint F (FDR-p)
FA	Lt. Caudate	**7.21 (0.015)**	**35.51 (<0.001)**	1.39 (0.239)
	Lt. Putamen	**8.45 (0.010)**	**20.65 (<0.001)**	**14.60 (0.002)**
	Lt. Pallidum	**5.62 (0.031)**	**34.22 (<0.001)**	**8.03 (0.013)**
	Lt. Hippocampus	**11.10 (0.006)**	3.70 (0.064)	**12.76 (0.002)**
	Lt. Amygdala	2.44 (0.918)	**9.69 (0.002)**	**10.37 (0.005)**
	Rt. Thalamus	0.60 (0.473)	**21.57 (<0.001)**	**7.21 (0.017)**
	Rt. Putamen	**9.52 (0.009)**	**94.38 (<0.001)**	4.41 (0.063)
	Rt. Pallidum	**11.04 (0.006)**	**66.52 (<0.001)**	**12.26 (0.002)**
	Rt. Hippocampus	**9.10 (0.009)**	**13.31 (<0.001)**	4.39 (0.063)
	Rt. Amygdala	**8.18 (0.010)**	3.30 (0.069)	3.87 (0.077)
MD	Lt. Hippocampus	0.69 (0.684)	**24.71 (<0.001)**	**10.84 (0.007)**
	Rt. Pallidum	**10.19 (0.020)**	0.74 (0.420)	0.22 (0.691)
	Rt. Hippocampus	0.03 (0.927)	**36.59 (<0.001)**	**14.20 (0.002)**

Values are F statistics with FDR-adjusted *p*-values in parentheses. Type III tests of fixed effects were obtained from longitudinal mixed-effects models. The group effect tests overall LP–FT differences within the longitudinal model, timepoint effect tests baseline-to-follow-up changes, and group-by-timepoint effect tests whether longitudinal changes differed between the LP and FT children. This table includes measures showing significant group effects or significant group-by-timepoint effects after FDR correction; complete Type III results for all subcortical ROIs FA and MD measures are provided in Supplementary Tables S7-5 and S7-6. Models included group, timepoint, and group-by-timepoint as fixed effects, with participant ID, family ID, and data collection site as random intercepts. Twin/triplet status and MRI scanner information were included as fixed-effect covariates. For neuroimaging outcomes, intracranial volume was included as a timepoint-specific covariate, with baseline ICV used for baseline observations and follow-up ICV used for follow-up observations. FDR correction was applied within each predefined outcome family and effect type. FDR-adjusted *p* < 0.05 was considered statistically significant. Bold values indicate these significant results. LP, late preterm; FT, full term; ROIs, region of interest; FDR, false discovery rate; FA, fractional anisotropy; MD, mean diffusivity; ICV, intracranial volume; Lt., left; Rt., right.

Post hoc simple-effects analyses were conducted for subcortical ROI metrics showing significant group-by-timepoint interactions. For subcortical FA, several ROIs showed no significant LP–FT differences at baseline but demonstrated significant LP–FT differences at follow-up. FT children showed significant follow-up-to-baseline decreases across these subcortical FA metrics, whereas LP children showed significant decreases only in selected regions. For subcortical MD, bilateral hippocampal metrics showed significant LP–FT differences at baseline but not at follow-up, with follow-up-to-baseline decreases observed in both groups (see Supplementary Table S9).

### 3.4 rs-fMRI

Type III tests from the longitudinal mixed-effects models showed significant timepoint effects in all the 12 functional networks after FDR correction. In contrast, no significant group effects or group-by-timepoint interactions were observed for any rs-fMRI network (see Supplementary Table S8).

### 3.5 Attrition analyses

Attrition analyses were conducted for brain volume and DTI domains. For brain volume, two-year follow-up data were available for 4,721 of 10,029 participants with baseline data, corresponding to an overall retention rate of 47.2%. Retention rates did not differ significantly between LP and FT children (48.3% vs. 46.9%, respectively; *p* = 0.391), and LP/FT group status was not associated with follow-up data availability in the adjusted logistic regression model (adjusted OR = 0.969, *p* = 0.676). For white matter tracts FA and MD, follow-up data were available for 4,105 of 9,160 participants (44.8%). Retention rates were similar in LP and FT children (46.3% vs. 44.6%; *p* = 0.324), and group status was not associated with follow-up data availability (adjusted OR = 0.984, *p* = 0.838). Comparable retention patterns were observed for cortical ROI FA and MD (4,104/9,159; *p* = 0.322) and subcortical ROI FA and MD (4,105/9,160; *p* = 0.324). Comparisons between completers and non-completers identified potential imbalance (absolute SMD ≥ 0.10) in some baseline characteristics and selected imaging measures. Thus, although attrition was not significantly associated with LP/FT group status, residual attrition bias cannot be excluded. Detailed results are provided in Supplementary Table S10.

## 4 Discussion

In this study, we examined the behavioral, cognitive, and multimodal neuroimaging outcomes in children born late preterm compared with children born full term using longitudinal data from the ABCD Study. Longitudinal mixed-effects models with Type III tests were used as the primary analytic framework. Overall, the findings did not reveal broad LP–FT differences in behavioral and emotional problems, cognitive function, cortical thickness, cortical surface area, or rs-fMRI within-network connectivity. Instead, significant group effects were observed in selected regional brain volumes and DTI metrics, and significant group-by-timepoint interactions were restricted to selected DTI metrics. These results suggest that LP-related neurodevelopmental differences during early adolescence are subtle, regionally specific, and most apparent in selected neuroimaging measures, particularly DTI metrics.

In the present community-based sample, late preterm birth was not associated with broad group-level differences in behavioral and cognitive findings. For behavioral and emotional problems, no significant group effects or group-by-timepoint interactions were observed, although somatic problems showed a timepoint effect. For cognitive function, a group effect was observed only for Flanker, and several cognitive measures showed timepoint effects, but no cognitive outcome showed a significant group-by-timepoint interaction. Thus, although several behavioral and cognitive measures changed from baseline to follow-up, the present results showed no evidence that these changes differed between the LP and FT children. This pattern is consistent with prior reports of mixed behavioral and cognitive outcomes in LP children (8, 11, 14, 15, 17) and suggests that broad functional measures may not fully capture subtle LP-related neurodevelopmental differences during early adolescence.

For sMRI, significant group effects were observed in six regional brain volume measures, whereas no significant timepoint effects or group-by-timepoint interactions were observed. Although selected regional brain volumes showed overall LP–FT differences within the longitudinal model, the present data did not provide evidence that the two-year volumetric change differed between the LP and FT children.

Ma et al., who used the same ABCD cohort to examine associations between gestational age, brain volume, and cognition in early adolescence (29), reported lower regional brain volumes among children born at lower gestational ages. Whereas the previous study categorized gestational age into multiple groups, including 34–35 weeks and 36 weeks separately, the present study combined children born at 34–36 weeks and compared them with those born full term at ≥40 weeks. Therefore, our findings complement prior ABCD-based work by showing that selected regional brain volume differences are detectable in early adolescence, even when LP children are examined as a clinically defined group, although we did not find evidence of differential two-year volumetric change.

DTI metrics showed the most prominent longitudinal findings. Significant group effects were observed in selected white matter tracts, cortical ROI, and subcortical ROI FA or MD metrics, whereas significant group-by-timepoint interactions were observed in selected DTI measures across these domains. *post hoc* simple-effects analyses suggested that many of these interactions were characterized by significant two-year decreases in FT children, whereas LP children showed smaller, non-significant, or less consistent changes. These *post hoc* analyses were descriptive and were used to clarify the direction of significant interaction terms rather than as independent primary tests.

These findings extend prior research on white matter development after preterm birth. Previous studies reported lower whole-brain white matter volume and FA in school-aged preterm children (53) and widespread microstructural alterations in moderate-to-late preterm infants at term-equivalent age (54). However, these studies examined broader preterm samples or infants in early life, and LP-specific DTI data during early adolescence remain limited.

ILF measures showed both FA and MD group effects, although in different hemispheres and diffusion metrics. This pattern suggests that the ILF may be one of the white matter tracts in which LP-related differences are relatively consistently detected within the longitudinal models. The ILF is a ventral temporo-occipital association pathway implicated in visual-semantic and language-related networks (55). However, the present findings should not be interpreted as evidence of ILF-specific impairment or altered language development, because broad LP–FT differences in cognitive outcomes were not observed. Moreover, the FA and MD findings differed by hemisphere and metric, and conventional DTI measures do not provide direct evidence of a single biological mechanism. FA and MD are sensitive to multiple aspects of tissue microstructure and do not provide direct evidence of a single biological mechanism (56). Therefore, the present findings should be interpreted as regionally specific differences in DTI metrics or two-year DTI metric changes, rather than as direct evidence of altered myelination, axonal organization, or white matter integrity.

Several limitations should be noted. First, attrition at the two-year follow-up was substantial. Retention and missing follow-up data are recognized challenges in large longitudinal neuroimaging cohorts, such as the ABCD Study (51). Although the LP/FT group status was not significantly associated with follow-up data availability, completers and non-completers differed in some observed baseline characteristics and selected imaging measures. Thus, attrition bias cannot be fully excluded.

Second, the present longitudinal analyses included only two timepoints, which limits the ability to characterize nonlinear developmental trajectories or determine whether the observed differences persist, attenuate, or become more pronounced later in adolescence. Future studies using additional follow-up waves will be needed to clarify longer-term developmental patterns.

Third, because this was an observational study, causal relationships between late preterm birth, neuroimaging measures, and behavioral or cognitive outcomes cannot be inferred. We also did not directly test whether neuroimaging findings mediated or predicted later functional outcomes. Therefore, the functional implications of the observed neuroimaging differences remain uncertain.

Fourth, detailed perinatal and neonatal factors were not fully incorporated into the analyses. These factors were not included in the primary adjusted models because several may lie on the causal pathway between gestational age and later neurodevelopmental outcomes, and their inclusion could result in overadjustment. However, this approach also means that residual confounding or effect modification by perinatal and neonatal factors cannot be fully excluded. In addition, gestational age was based on retrospective parent/caregiver report rather than obstetric medical records. Therefore, misclassification of gestational-age category cannot be excluded, particularly near category boundaries. To reduce potential overlap between the groups, we used a conservative grouping strategy by comparing children born late preterm, 34–36 weeks, with those born full term at ≥40 weeks and excluding children born at 37–39 weeks from the primary comparison.

Finally, the generalizability of the findings should be considered carefully. Although the ABCD Study provides a large, standardized, multi-site cohort conducted in the United States, it was not designed as a perinatal birth cohort specifically sampling LP children. Therefore, LP children included in these analyses may not fully represent all children born late preterm, particularly those with substantial neonatal morbidity, prolonged neonatal hospitalization, or complex perinatal histories. Because the cohort was recruited within the United States, the findings should also be interpreted in the context of the United States demographic, medical, healthcare, and sociocultural factors and may not be directly generalizable to LP children in other countries or healthcare systems.

In conclusion, longitudinal mixed-effects analyses showed that late preterm birth was associated with selected group differences in regional brain volume and DTI metrics, whereas group-by-timepoint interactions were restricted to DTI metrics. Behavioral, cognitive, cortical morphometric, and rs-fMRI outcomes did not show evidence of broad differential longitudinal changes between the LP and FT children. These findings suggest that LP-related neurodevelopmental differences during early adolescence may be subtle and regionally specific, with DTI metrics being sensitive to differences in two-year developmental change. The findings should be interpreted cautiously, owing to the follow-up attrition, two-timepoint design, and observational nature of the study.

## Data Availability

The original contributions presented in the study are included in the article/Supplementary Material, further inquiries can be directed to the corresponding author.
