# Supplementary material for: Neurodevelopment and brain maturation in late preterm children: insights from the adolescent brain cognitive development (ABCD) study
Source: Front Pediatr. 2026 Jul 20;14:1827902. doi: 10.3389/fped.2026.1827902 (PMC13429664; doi:10.3389/fped.2026.1827902)
Supplement: Supplementary file 1 [file Supplementaryfile1.pdf]

Supplementary Material

1 Supplementary Methods

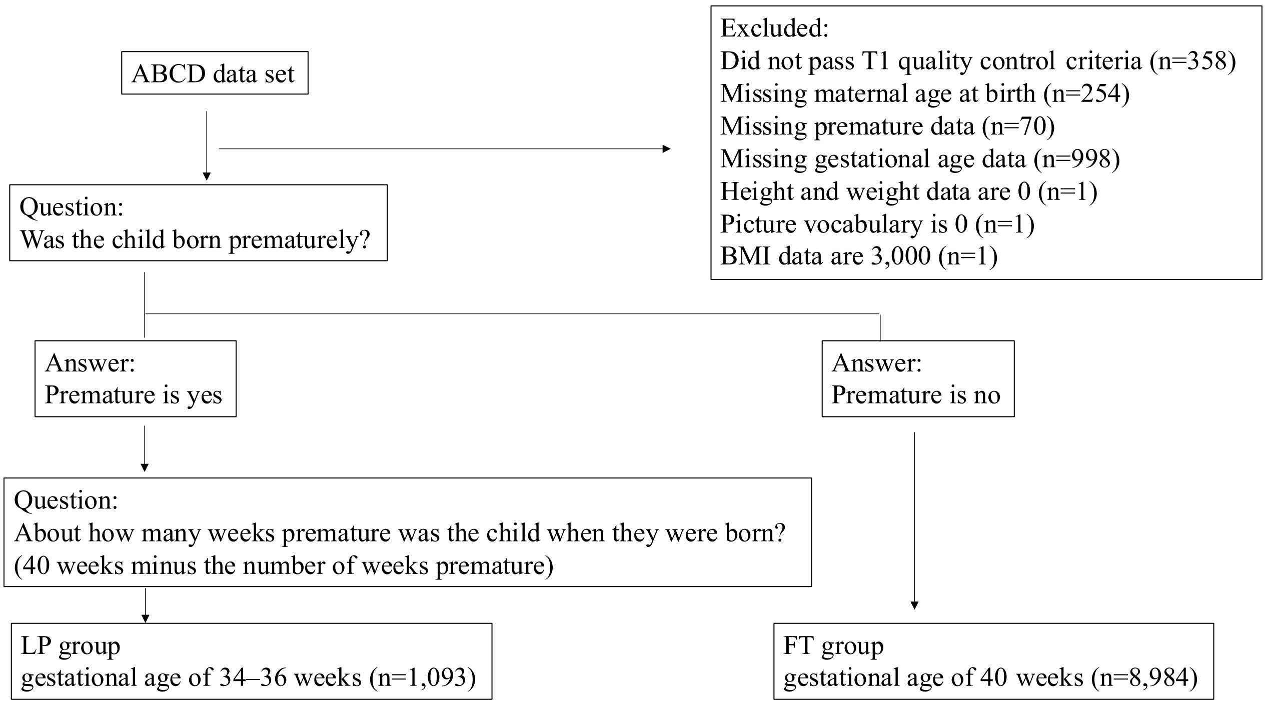

Figure S1. Adolescents enrolled in the ABCD Study at baseline (9–11 years).
Flowchart for classifying children into the LP and FT groups based on gestational age and the exclusion criteria for analysis of behavioral and emotional problems, cognitive function, and brain structure.
BMI, body mass index; FT, full-term; LP, late preterm; ABCD, Adolescent Brain Cognitive Development

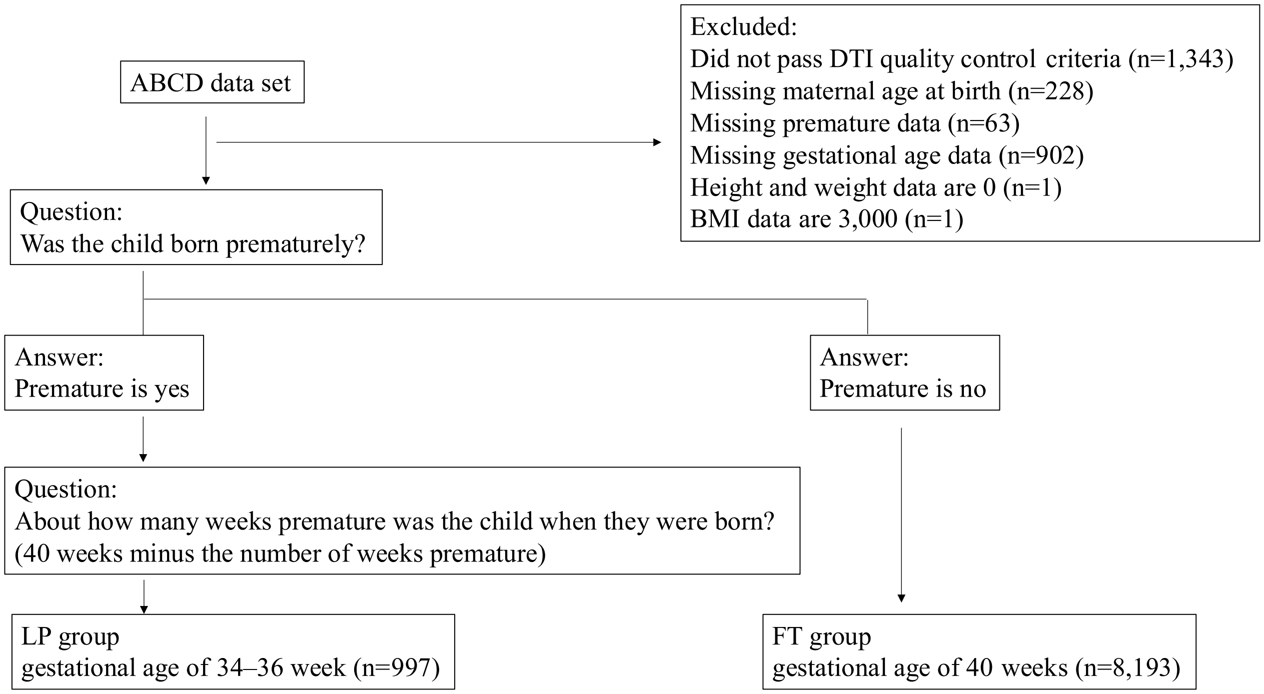

Figure S2. Adolescents enrolled in the ABCD Study at baseline (9–11 years).
Flowchart for classifying children into the LP and FT groups based on gestational age and the exclusion criteria for DTI analysis.
BMI, body mass index; DTI, diffusion tensor imaging; FT, full-term; LP, late preterm; ABCD, Adolescent Brain Cognitive Development

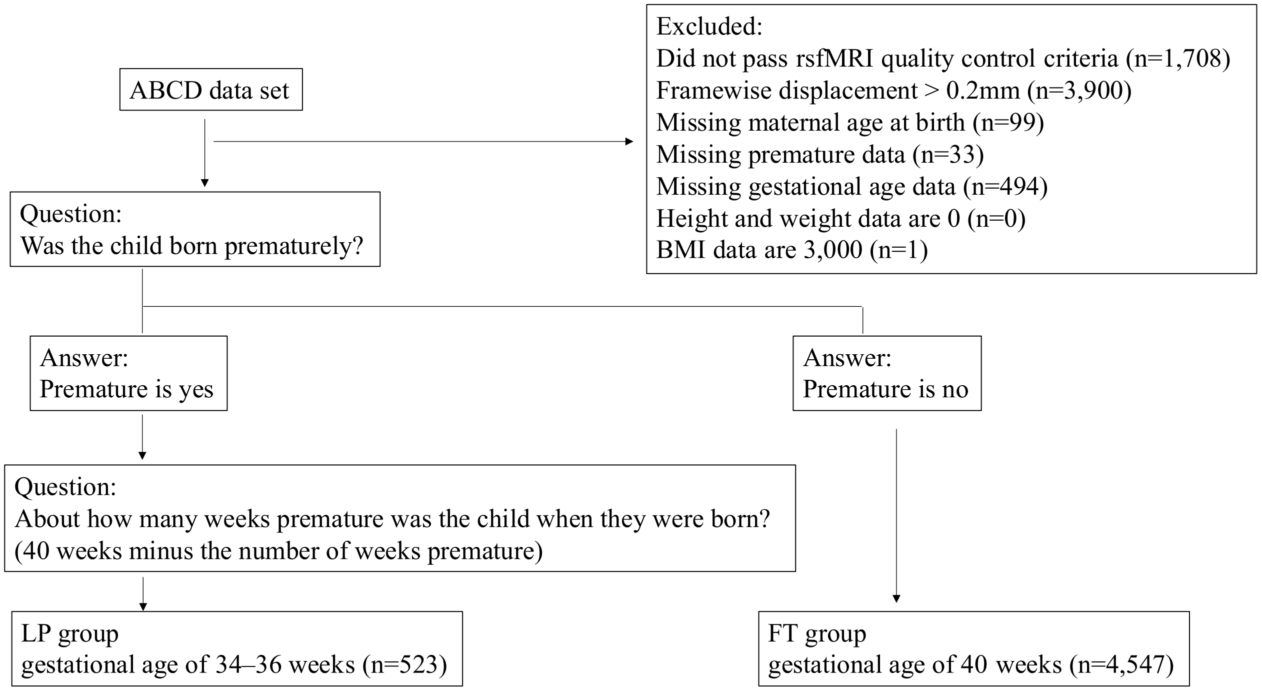

Figure S3. Adolescents enrolled in the ABCD Study at baseline (9–11 years).
Flowchart for classifying children into the LP and FT groups based on gestational age and the exclusion criteria for rs-fMRI analysis.
BMI, body mass index; FT, full-term; LP, late preterm; rs-fMRI, resting-state functional magnetic resonance imaging; ABCD, Adolescent Brain Cognitive Development

Child Behavior Checklist (CBCL)

The CBCL is a parent-reported questionnaire for assessing behavioral and emotional problems in children (1). The CBCL comprises 113 questions that assess behavior aspects in children aged >6 months, with each item scored on a 3-point Likert scale (0=not true, 1=somewhat or sometimes true, and 2=very true or often true). Additionally, it has two empirically derived broadband scales representing internalizing and externalizing problems, eight empirically derived syndrome scales indicating different patterns of comorbid emotional and behavioral problems, and six Diagnostic & Statistical Manual of Mental Disorders (DSM)-oriented scales derived through expert consensus. The six DSM-oriented scales include Depression, Anxiety disorder, Somatic problem, attention deficit hyperactivity disorder (ADHD), Oppositional defiant disorder, and Conduct disorder. Each DSM-oriented scale reflects a broad emotional or behavioral problem that corresponds to a broad DSM diagnostic category. The higher the score, the more severe the problem. All scores are recorded as t-scores, with T-scores ≥70, 65–69, and <64 on the six syndrome scales indicating clinical symptomology, cause for concern, and normality, respectively.

National Institutes of Health Toolbox Tasks

The National Institutes of Health Toolbox Tasks were performed using an iPad-based program in the ABCD Study (2). In the flanker inhibitory control and attention task, participants were instructed to

indicate the left–right orientation of a central arrow flanked by two arrows pointing in the same (congruent) or different (incongruent) directions. Twenty trials were presented, with the flanking arrows alternating randomly between congruence and incongruence with the middle arrow. The score was generated by combining response time and accuracy vectors. In the dimensional change card sort task, participants were presented with objects at the bottom of the screen. Participants were asked to sort a third object presented in the middle of the screen based on either color or shape to match one of the two objects at the bottom of the screen. They indicated their response using touch input. The total score was based on both accuracy and reaction time. In the pattern comparison processing speed task, participants were instructed to use their dominant hand to tap "yes" if the stimuli presented on the screen were the same and "no" if they were not. Stimuli were presented in succession over 90 s, with the participant responding to as many stimuli as possible within this period. The score was based on the number of items (out of a possible 130) answered correctly in the timeframe. In the picture sequence memory task, images and verbal statements of events (e.g., "going to the park") were presented sequentially and assigned corresponding positions on the screen. Subsequently, images were presented scrambled in the screen center, and participants were instructed to replicate the sequence by dragging the images to their appropriate positions. Sequences varied from 6 to 18 pictures, based on the chronological age of the participants. The score was generated based on the number of adjacent image pairs placed in the correct order for each trial. In the list sorting working memory task, participants were presented with a series of pictures of animals or foods of different sizes. Participants were asked to repeat back to the experimenter the items that were presented, in order from smallest to largest. Participants had two chances to provide a correct answer at each list length and continued to the next length—up to a maximum length of seven—if they correctly answered at least one of the items. In the picture vocabulary task, participants were instructed to select an image from a set of four that corresponded to a read-out word. Twenty-five trials were represented, and the scores were based on the number of correct responses. In the oral reading recognition task, participants were instructed to read out words that were presented on the screen. The participants received one of the four forms (form 1, 70 items; form 2, 101 items; form 3, 120 items; and form 4, 125 items) and attempted items until they either completed the prescribed number of items for their form or mispronounced 10 words in a row. The scores were based on the number of correct responses.

2 Supplementary Results

Table S1. Significant findings from descriptive baseline LP–FT comparisons

Region/Tracts/ ROIs		estimate	95%CI	T value	Raw p	FDR-p
Volume	Rt. Pars orbitalis	-0.044	[-0.065, -0.024]	-4.182	<0.001	**0.002**
White matter tracts MD	Lt. Inferior longitudinal fasciculus	-0.042	[-0.065, -0.019]	-3.600	<0.001	**0.011**
	Rt. Inferior longitudinal fasciculus	-0.037	[-0.061, -0.013]	-3.053	<0.001	**0.040**
	Rt. Temporal superior longitudinal fasciculus	-0.034	[-0.057, -0.010]	-2.809	0.005	**0.044**
	Rt. Superior corticostriate-frontal cortex	-0.033	[-0.056, -0.010]	-2.841	0.005	**0.044**
Cortical FA	Lt. Peri calcarine	0.045	[0.021, 0.069]	3.705	<0.001	**0.005**
	Lt. Supra marginal	0.032	[0.011, 0.053]	2.953	0.003	**0.036**
	Lt. Transverse temporal	0.042	[0.019, 0.066]	3.505	<0.001	**0.008**
	Rt. Lingual	0.052	[0.029, 0.076]	4.367	<0.001	**<0.001**
	Rt. Middle temporal	0.035	[0.013, 0.057]	3.121	0.002	**0.025**
	Rt. Peri calcarine	0.045	[0.022, 0.069]	3.775	<0.001	**0.005**
Subcortical MD	Rt. Pallidum	-0.036	[-0.058, -0.014]	-3.203	0.001	**0.019**

<footnote>

Baseline analyses were conducted as descriptive time-point-specific analyses, but not as the primary test of longitudinal effects. Linear mixed-effects models included birth group as the primary fixed effect, with family ID and data collection site as random intercepts. Twin/triplet status and MRI scanner information were included as fixed-effect covariates. Models were adjusted for demographic and socioeconomic covariates, pubertal score, body mass index, and baseline intracranial volume. Estimates represent adjusted LP–FT differences based on standardized outcomes; negative estimates indicate lower values in LP children relative to FT children. P values were adjusted using the Benjamini–Hochberg FDR procedure within each predefined outcome family. Only FDR-significant findings are shown. Bold values indicate these significant results. LP, late preterm; FT, full term; CI, confidence interval; FDR, false discovery rate; ROI, region of interest; FA, fractional anisotropy; MD, mean diffusivity; Lt., left; Rt., right; MRI, magnetic resonance imaging.

Table S2. Complete Type III longitudinal mixed-effects model results for behavioral and emotional problem outcomes

Outcome	Effect	F	raw_p	FDR_p
Depress	Group	4.73	0.03	0.057
	Timepoint	0.10	0.756	0.756
	Group x timepoint	0.57	0.451	0.667
anxdisord	Group	4.56	0.033	0.057
	Timepoint	3.35	0.067	0.163
	Group x timepoint	2.05	0.153	0.667
somaticpr	Group	4.31	0.038	0.057
	Timepoint	9.98	0.002	**0.010**
	Group x timepoint	0.35	0.556	0.667
ADHD	Group	5.65	0.017	0.057
	Timepoint	0.43	0.512	0.614
	Group x timepoint	0.81	0.367	0.667
opposite	Group	2.83	0.093	0.102
	Timepoint	3.04	0.081	0.163
	Group x timepoint	0.57	0.451	0.667
Coduct	Group	2.67	0.102	0.102
	Timepoint	0.46	0.498	0.614
	Group x timepoint	0.13	0.723	0.723

<Footnote>

Type III tests of fixed effects were obtained from longitudinal mixed-effects models. The group effect tests overall LP–FT differences within the longitudinal model, timepoint effect tests baseline-to-follow-up changes, and group-by-timepoint effect tests whether longitudinal changes differed between the LP and FT children. Models included group, timepoint, and group-by-timepoint as fixed effects, with participant ID, family ID, and data collection site as random intercepts. Twin/triplet status and MRI scanner information were included as fixed-effect covariates. F values are Type III F statistics obtained from longitudinal mixed-effects models. Raw p values and FDR-adjusted p values are shown. FDR correction was applied within the corresponding outcome family. FDR-adjusted $p < 0.05$ was considered statistically significant. Bold values indicate these significant results. CBCL T-scores were based on the DSM-5-oriented scales. Data are presented as the mean ± standard deviation for each measure. LP, late preterm; MRI, magnetic resonance imaging; FT, full term; FDR, false discovery rate; CBCL, Child Behavior Checklist; DSM-5, Diagnostic and Statistical Manual of Mental Disorders; Anxdisord, anxiety disorder; Somaticpr, somatic problem; ADHD, attention-deficit/hyperactivity disorder; Opposit, oppositional defiant disorder; Conduct, conduct disorder.

Table S3. Complete Type III longitudinal mixed-effects model results for cognitive function outcomes

Outcome	Effect	F	raw_p	FDR_p
Picvocab	Group	0.002	0.963	0.963
	Timepoint	134.28	<0.001	**<0.001**
	Group x timepoint	0.01	0.932	0.932
Flanker	Group	7.88	0.005	**0.045**
	Timepoint	6.34	0.012	**0.015**
	Group x timepoint	2.58	0.108	0.486
List	Group	1.15	0.29	0.500
	Timepoint	0.01	0.932	0.932
	Group x timepoint	1.26	0.269	0.669
DCCS	Group	0.35	0.555	0.624
	Timepoint	3.59	0.064	0.072
	Group x timepoint	0.04	0.84	0.932
Pattern	Group	3.35	0.067	0.202
	Timepoint	49.87	<0.001	**<0.001**
	Group x timepoint	0.19	0.666	0.932
Picture	Group	5.59	0.018	0.081
	Timepoint	12.75	<0.001	**<0.001**
	Group x timepoint	1.09	0.297	0.669
Reading	Group	0.74	0.389	0.500
	Timepoint	48.04	<0.001	**<0.001**
	Group x timepoint	2.99	0.084	0.486
Short_memory	Group	1.62	0.204	0.458
	Timepoint	41.57	<0.001	**<0.001**
	Group x timepoint	0.02	0.893	0.932
Long_memory	Group	0.82	0.367	0.500
	Timepoint	53.58	<0.001	**<0.001**
	Group x timepoint	0.06	0.808	0.932

<Footnote>

Type III tests of fixed effects were obtained from longitudinal mixed-effects models. Cognitive outcomes included seven NIH Toolbox measures and two RAVLT measures. The group effect tests overall LP–FT differences within the longitudinal model, timepoint effect tests baseline-to-follow-up change, and group-by-timepoint effect tests whether longitudinal change differed between the LP and FT children. Models included group, timepoint, and group-by-timepoint as fixed effects, with

participant ID, family ID, and data collection site as random intercepts. Twin/triplet status was included as a fixed-effect covariate. F values are Type III F statistics obtained from longitudinal mixed-effects models. Raw and FDR-adjusted p values are shown. FDR correction was applied within the cognitive outcome family and separately for each effect type. FDR-adjusted $p < 0.05$ was considered statistically significant. Bold values indicate these significant results. NIH, National Institutes of Health; RAVLT, the Rey Auditory Verbal Learning Test; LP, late preterm; FT, full term; FDR, false discovery rate; Picvocab, picture vocabulary; Flanker, flanker inhibitory control and attention; List, list sorting working memory; DCCS, dimensional change card sort; Pattern, pattern-comparison-processing speed; Picture, picture sequence memory; Reading, oral reading recognition.

Table S4. Complete Type III longitudinal mixed-effects model results for Brain volume measures

Regions	Effect	F	raw_p	FDR_p
Left				
Banks of superior temporal sulcus	Group	5.35	0.021	0.106
	Timepoint	0.05	0.828	0.965
	Group x timepoint	0.97	0.324	0.919
Caudal anterior cingulate	Group	0.003	0.958	0.958
	Timepoint	6.88	0.009	0.179
	Group x timepoint	0.37	0.545	0.919
Caudal middle frontal	Group	0.28	0.598	0.732
	Timepoint	0.31	0.578	0.910
	Group x timepoint	0.13	0.723	0.919
Cuneus	Group	0.21	0.649	0.760
	Timepoint	0.09	0.758	0.953
	Group x timepoint	0.64	0.423	0.919
Entorhinal	Group	0.71	0.400	0.597
	Timepoint	5.42	0.020	0.308
	Group x timepoint	0.48	0.489	0.919
Fusiform	Group	7.44	0.006	0.075
	Timepoint	0.03	0.863	0.965
	Group x timepoint	0.001	0.972	0.973
Inferior parietal	Group	6.69	0.01	0.100
	Timepoint	0.18	0.673	0.936
	Group x timepoint	0.06	0.801	0.930
Inferior temporal	Group	0.10	0.751	0.832
	Timepoint	0.03	0.859	0.965
	Group x timepoint	1.54	0.214	0.919
Isthmus cingulate	Group	0.95	0.330	0.536
	Timepoint	0.25	0.617	0.910
	Group x timepoint	0.18	0.67	0.919
Lateral occipital	Group	0.88	0.348	0.539
	Timepoint	0.18	0.673	0.936
	Group x timepoint	0.73	0.392	0.919
Lateral orbitofrontal	Group	0.66	0.415	0.608
	Timepoint	6.96	0.008	0.179
	Group x timepoint	1.45	0.228	0.919

Lingual	Group	0.94	0.333	0.536
	Timepoint	0.93	0.335	0.767
	Group x timepoint	0.79	0.375	0.919
Medial orbitofrontal	Group	1.50	0.22	0.420
	Timepoint	3.25	0.071	0.489
	Group x timepoint	2.73	0.099	0.919
Middle temporal	Group	6.26	0.012	0.100
	Timepoint	0.64	0.422	0.787
	Group x timepoint	7.80	0.005	0.431
Parahippocampal	Group	1.19	0.276	0.491
	Timepoint	2.82	0.093	0.489
	Group x timepoint	0.19	0.664	0.919
Paracentral	Group	4.50	0.034	0.147
	Timepoint	1.54	0.215	0.716
	Group x timepoint	0.15	0.698	0.919
Pars opercularis	Group	2.53	0.112	0.306
	Timepoint	0.08	0.772	0.953
	Group x timepoint	0.57	0.449	0.919
Pars orbitalis	Group	5.66	0.017	0.106
	Timepoint	4.10	0.043	0.441
	Group x timepoint	0.21	0.643	0.919
Pars triangularis	Group	5.38	0.020	0.106
	Timepoint	2.34	0.126	0.518
	Group x timepoint	0.84	0.359	0.919
Peri calcarine	Group	0.48	0.489	0.625
	Timepoint	0.004	0.948	0.984
	Group x timepoint	1.04	0.308	0.919
Post central	Group	8.54	0.003	**0.048**
	Timepoint	0.49	0.485	0.839
	Group x timepoint	0.04	0.835	0.930
Posterior cingulate	Group	1.89	0.169	0.396
	Timepoint	0.04	0.835	0.965
	Group x timepoint	0.10	0.748	0.919
Precentral	Group	5.90	0.015	0.104
	Timepoint	0.83	0.363	0.767
	Group x timepoint	0.54	0.462	0.919

Precuneus	Group	0.40	0.529	0.657
	Timepoint	1.25	0.264	0.745
	Group x timepoint	1.00	0.318	0.919
Rostral anterior cingulate	Group	2.01	0.156	0.388
	Timepoint	1.44	0.231	0.716
	Group x timepoint	0.48	0.486	0.919
Rostral middle frontal	Group	1.65	0.199	0.414
	Timepoint	0.61	0.435	0.793
	Group x timepoint	0.82	0.364	0.919
Superior frontal	Group	5.58	0.018	0.106
	Timepoint	4.94	0.026	0.308
	Group x timepoint	0.70	0.402	0.919
Superior parietal	Group	1.35	0.245	0.457
	Timepoint	0.74	0.391	0.767
	Group x timepoint	0.30	0.582	0.919
Superior temporal	Group	0.26	0.611	0.737
	Timepoint	9.51	0.002	0.168
	Group x timepoint	2.22	0.136	0.919
Supra marginal	Group	4.09	0.043	0.168
	Timepoint	2.86	0.091	0.489
	Group x timepoint	0.002	0.968	0.973
Frontal pole	Group	0.04	0.839	0.871
	Timepoint	0.85	0.358	0.767
	Group x timepoint	0.48	0.487	0.919
Temporal pole	Group	0.005	0.944	0.956
	Timepoint	1.02	0.313	0.767
	Group x timepoint	2.17	0.141	0.919
Transverse temporal	Group	0.11	0.74	0.832
	Timepoint	1.19	0.275	0.753
	Group x timepoint	0.004	0.952	0.973
Insula	Group	6.23	0.013	0.100
	Timepoint	0.04	0.846	0.965
	Group x timepoint	1.03	0.309	0.919
Thalamus	Group	0.07	0.786	0.860
	Timepoint	1.50	0.221	0.716
	Group x timepoint	0.001	0.973	0.973

Caudate	Group	0.24	0.624	0.741
	Timepoint	0.47	0.491	0.839
	Group x timepoint	1.90	0.168	0.919
Putamen	Group	0.94	0.333	0.536
	Timepoint	0.11	0.735	0.942
	Group x timepoint	0.08	0.783	0.930
Pallidum	Group	1.20	0.273	0.491
	Timepoint	0.02	0.885	0.965
	Group x timepoint	0.88	0.347	0.919
Hippocampus	Group	0.91	0.34	0.536
	Timepoint	0.12	0.726	0.942
	Group x timepoint	2.94	0.086	0.919
Amygdala	Group	1.63	0.202	0.414
	Timepoint	0.25	0.619	0.910
	Group x timepoint	0.64	0.424	0.919
Accumbens area	Group	3.36	0.067	0.204
	Timepoint	3.25	0.072	0.489
	Group x timepoint	0.22	0.641	0.919
Right				
Banks of superior temporal sulcus	Group	6.12	0.013	0.100
	Timepoint	0.97	0.324	0.767
	Group x timepoint	0.01	0.934	0.973
Caudal anterior cingulate	Group	1.92	0.166	0.396
	Timepoint	0.55	0.459	0.819
	Group x timepoint	0.10	0.751	0.919
Caudal middle frontal	Group	1.66	0.198	0.414
	Timepoint	0.02	0.893	0.965
	Group x timepoint	0.14	0.708	0.919
Cuneus	Group	3.42	0.064	0.204
	Timepoint	0.03	0.852	0.965
	Group x timepoint	0.29	0.592	0.919
Entorhinal	Group	2.02	0.156	0.388
	Timepoint	0.23	0.629	0.910
	Group x timepoint	1.25	0.264	0.919
Fusiform	Group	9.24	0.002	**0.048**

	Timepoint	0.01	0.904	0.965
	Group x timepoint	2.17	0.141	0.919
Inferior parietal	Group	4.13	0.042	0.168
	Timepoint	0.00	0.987	0.997
	Group x timepoint	0.37	0.545	0.919
Inferior temporal	Group	0.53	0.466	0.615
	Timepoint	0.70	0.403	0.769
	Group x timepoint	6.32	0.012	0.492
Isthmus cingulate	Group	4.01	0.045	0.169
	Timepoint	0.002	0.962	0.986
	Group x timepoint	0.13	0.722	0.919
Lateral occipital	Group	0.59	0.442	0.615
	Timepoint	0.81	0.368	0.767
	Group x timepoint	0.42	0.516	0.919
Lateral orbitofrontal	Group	2.04	0.154	0.388
	Timepoint	1.33	0.249	0.730
	Group x timepoint	0.43	0.51	0.919
Lingual	Group	0.05	0.815	0.870
	Timepoint	2.11	0.146	0.571
	Group x timepoint	0.15	0.702	0.919
Medial orbitofrontal	Group	1.50	0.22	0.420
	Timepoint	3.18	0.075	0.489
	Group x timepoint	1.35	0.246	0.919
Middle temporal	Group	13.9	<0.001	**0.008**
	Timepoint	0.14	0.705	0.942
	Group x timepoint	3.15	0.076	0.919
Parahippocampal	Group	0.54	0.461	0.615
	Timepoint	2.72	0.099	0.489
	Group x timepoint	1.00	0.317	0.919
Paracentral	Group	3.29	0.07	0.204
	Timepoint	0.73	0.393	0.767
	Group x timepoint	0.49	0.484	0.919
Pars opercularis	Group	2.95	0.086	0.242
	Timepoint	0.32	0.574	0.910
	Group x timepoint	0.08	0.782	0.930
Pars orbitalis	Group	16.61	<0.001	**0.004**

	Timepoint	0.39	0.534	0.894
	Group x timepoint	0.85	0.357	0.919
Pars triangularis	Group	5.14	0.023	0.113
	Timepoint	5.20	0.023	0.308
	Group x timepoint	0.04	0.839	0.930
Peri calcarine	Group	0.47	0.495	0.625
	Timepoint	0.08	0.779	0.953
	Group x timepoint	0.40	0.525	0.919
Post central	Group	1.58	0.209	0.417
	Timepoint	0.12	0.726	0.942
	Group x timepoint	1.64	0.20	0.919
Posterior cingulate	Group	0.59	0.442	0.615
	Timepoint	0.16	0.689	0.941
	Group x timepoint	0.13	0.713	0.919
Precentral	Group	1.83	0.176	0.402
	Timepoint	2.34	0.126	0.518
	Group x timepoint	1.45	0.229	0.919
Precuneus	Group	0.12	0.729	0.832
	Timepoint	7.60	0.006	0.179
	Group x timepoint	0.18	0.67	0.919
Rostral anterior cingulate	Group	3.62	0.057	0.195
	Timepoint	2.49	0.115	0.518
	Group x timepoint	0.36	0.548	0.919
Rostral middle frontal	Group	3.35	0.067	0.204
	Timepoint	0.3	0.582	0.910
	Group x timepoint	0.19	0.662	0.919
Superior frontal	Group	3.72	0.054	0.191
	Timepoint	1.57	0.21	0.716
	Group x timepoint	0.11	0.737	0.919
Superior parietal	Group	4.86	0.027	0.125
	Timepoint	0.23	0.633	0.910
	Group x timepoint	0.72	0.398	0.919
Superior temporal	Group	0.02	0.895	0.918
	Timepoint	1.41	0.236	0.716
	Group x timepoint	0.18	0.673	0.919
Supra marginal	Group	11.25	<0.001	**0.022**

	Timepoint	2.69	0.101	0.489
	Group x timepoint	0.30	0.582	0.919
Frontal pole	Group	1.75	0.187	0.413
	Timepoint	0.26	0.61	0.910
	Group x timepoint	0.72	0.397	0.919
Temporal pole	Group	0.05	0.829	0.871
	Timepoint	1.11	0.292	0.767
	Group x timepoint	0.03	0.866	0.947
Transverse temporal	Group	1.16	0.282	0.492
	Timepoint	2.90	0.088	0.489
	Group x timepoint	1.31	0.253	0.919
Insula	Group	8.85	0.003	**0.048**
	Timepoint	0.79	0.375	0.767
	Group x timepoint	0.05	0.822	0.930
Thalamus	Group	0.76	0.384	0.583
	Timepoint	0.85	0.355	0.767
	Group x timepoint	0.26	0.607	0.919
Caudate	Group	0.10	0.747	0.832
	Timepoint	0.77	0.379	0.767
	Group x timepoint	1.92	0.166	0.919
Putamen	Group	0.62	0.431	0.615
	Timepoint	0.01	0.906	0.965
	Group x timepoint	0.05	0.822	0.93
Pallidum	Group	0.97	0.325	0.536
	Timepoint	1.52	0.218	0.716
	Group x timepoint	0.004	0.947	0.973
Hippocampus	Group	0.05	0.817	0.870
	Timepoint	0.01	0.933	0.981
	Group x timepoint	0.02	0.88	0.949
Amygdala	Group	0.52	0.472	0.615
	Timepoint	0.00	0.997	0.997
	Group x timepoint	0.73	0.394	0.919
Accumbens area	Group	0.53	0.467	0.615
	Timepoint	3.62	0.057	0.489
	Group x timepoint	0.43	0.513	0.919

<footnote>

Type III tests of fixed effects were obtained from longitudinal mixed-effects models. The group effect tests overall LP–FT differences within the longitudinal model, timepoint effect tests baseline-to-follow-up change, and group-by-timepoint effect tests whether longitudinal change differed between the LP and FT children. Models included group, timepoint, and group-by-timepoint as fixed effects, with participant ID, family ID, and data collection site as random intercepts. Twin/triplet status and MRI scanner information were included as fixed-effect covariates. For neuroimaging outcomes, intracranial volume was included as a timepoint-specific covariate. F values are Type III F statistics from longitudinal mixed-effects models. Raw and FDR-adjusted p values are shown. FDR correction was applied within the corresponding outcome family. FDR-adjusted $p < 0.05$ was considered statistically significant. Bold values indicate these significant results. LP, late preterm; FT, full term; FDR, false discovery rate; MRI, magnetic resonance imaging

Table S5. Complete Type III longitudinal mixed-effects model results for cortical thickness measures

Regions	Effect	F	raw_p	FDR_p
Left				
Banks of superior temporal sulcus	Group	7.67	0.006	0.128
	Timepoint	4.87	0.027	0.180
	Group x timepoint	0.91	0.34	0.746
Caudal anterior cingulate	Group	1.51	0.219	0.595
	Timepoint	0.06	0.807	0.946
	Group x timepoint	0.09	0.767	0.949
Caudal middle frontal	Group	0.44	0.506	0.752
	Timepoint	5.33	0.021	0.175
	Group x timepoint	0.13	0.721	0.948
Cuneus	Group	5.23	0.022	0.303
	Timepoint	0.51	0.477	0.748
	Group x timepoint	0.04	0.837	0.949
Entorhinal	Group	0.55	0.46	0.752
	Timepoint	1.45	0.229	0.487
	Group x timepoint	3.43	0.064	0.479
Fusiform	Group	0.03	0.872	0.920
	Timepoint	3.60	0.058	0.221
	Group x timepoint	3.66	0.056	0.474
Inferior parietal	Group	2.34	0.126	0.496
	Timepoint	1.66	0.198	0.448
	Group x timepoint	1.22	0.269	0.710
Inferior temporal	Group	0.15	0.698	0.920
	Timepoint	6.13	0.013	0.129
	Group x timepoint	3.72	0.054	0.474
Isthmus cingulate	Group	0.04	0.849	0.920
	Timepoint	0.63	0.429	0.729
	Group x timepoint	0.03	0.857	0.955
Lateral occipital	Group	4.44	0.035	0.304
	Timepoint	3.22	0.073	0.235
	Group x timepoint	0.61	0.436	0.809
Lateral orbitofrontal	Group	0.48	0.488	0.752
	Timepoint	1.25	0.264	0.545
	Group x timepoint	0.05	0.829	0.949

Lingual	Group	0.03	0.865	0.920
	Timepoint	9.72	0.002	0.093
	Group x timepoint	4.48	0.034	0.432
Medial orbitofrontal	Group	0.35	0.552	0.782
	Timepoint	0.24	0.622	0.811
	Group x timepoint	1.77	0.183	0.651
Middle temporal	Group	2.96	0.085	0.414
	Timepoint	0.42	0.517	0.748
	Group x timepoint	10.52	0.001	0.081
Parahippocampal	Group	0.48	0.491	0.752
	Timepoint	0.70	0.403	0.716
	Group x timepoint	0.12	0.725	0.948
Paracentral	Group	0.66	0.416	0.752
	Timepoint	5.16	0.023	0.175
	Group x timepoint	0.01	0.911	0.980
Pars opercularis	Group	0.11	0.737	0.920
	Timepoint	1.73	0.189	0.443
	Group x timepoint	0.59	0.442	0.809
Pars orbitalis	Group	0.88	0.349	0.752
	Timepoint	0.75	0.385	0.716
	Group x timepoint	0.63	0.426	0.809
Pars triangularis	Group	0.72	0.398	0.752
	Timepoint	2.38	0.123	0.334
	Group x timepoint	1.69	0.194	0.651
Peri calcarine	Group	3.76	0.052	0.387
	Timepoint	2.26	0.133	0.348
	Group x timepoint	0.63	0.426	0.809
Post central	Group	0.42	0.519	0.752
	Timepoint	0.39	0.532	0.753
	Group x timepoint	0.19	0.66	0.936
Posterior cingulate	Group	0.52	0.472	0.752
	Timepoint	0.23	0.632	0.811
	Group x timepoint	6.23	0.013	0.286
Precentral	Group	0.84	0.359	0.752
	Timepoint	8.07	0.005	0.093
	Group x timepoint	0.15	0.696	0.948

Precuneus	Group	7.90	0.005	0.128
	Timepoint	4.29	0.038	0.199
	Group x timepoint	0.001	0.975	0.980
Rostral anterior cingulate	Group	1.99	0.158	0.513
	Timepoint	1.02	0.312	0.606
	Group x timepoint	0.35	0.553	0.850
Rostral middle frontal	Group	3.24	0.072	0.387
	Timepoint	0.23	0.631	0.811
	Group x timepoint	0.41	0.523	0.850
Superior frontal	Group	1.07	0.301	0.712
	Timepoint	6.24	0.012	0.129
	Group x timepoint	0.001	0.980	0.980
Superior parietal	Group	0.41	0.52	0.752
	Timepoint	2.47	0.116	0.334
	Group x timepoint	0.06	0.812	0.949
Superior temporal	Group	0.00	0.999	0.999
	Timepoint	2.00	0.158	0.397
	Group x timepoint	2.98	0.084	0.479
Supra marginal	Group	1.27	0.26	0.680
	Timepoint	3.91	0.048	0.216
	Group x timepoint	0.55	0.456	0.809
Frontal pole	Group	0.51	0.474	0.752
	Timepoint	0.01	0.907	0.963
	Group x timepoint	1.64	0.200	0.651
Temporal pole	Group	2.19	0.139	0.496
	Timepoint	0.17	0.681	0.842
	Group x timepoint	3.10	0.079	0.479
Transverse temporal	Group	0.04	0.850	0.920
	Timepoint	0.02	0.891	0.963
	Group x timepoint	0.004	0.950	0.980
Insula	Group	2.24	0.134	0.496
	Timepoint	0.00	0.979	0.981
	Group x timepoint	1.64	0.201	0.651
Right				
Banks of superior temporal sulcus	Group	4.41	0.036	0.304
	Timepoint	0.44	0.505	0.748

	Group x timepoint	0.05	0.818	0.949
Caudal anterior cingulate	Group	0.07	0.798	0.920
	Timepoint	0.14	0.713	0.850
	Group x timepoint	0.96	0.328	0.744
Caudal middle frontal	Group	2.59	0.107	0.479
	Timepoint	3.58	0.058	0.221
	Group x timepoint	1.11	0.292	0.710
Cuneus	Group	5.54	0.019	0.303
	Timepoint	0.01	0.923	0.965
	Group x timepoint	1.44	0.230	0.657
Entorhinal	Group	0.02	0.880	0.920
	Timepoint	0.001	0.981	0.981
	Group x timepoint	0.05	0.827	0.949
Fusiform	Group	1.82	0.178	0.525
	Timepoint	0.44	0.507	0.748
	Group x timepoint	1.12	0.291	0.710
Inferior parietal	Group	0.02	0.902	0.929
	Timepoint	1.13	0.288	0.576
	Group x timepoint	1.76	0.184	0.651
Inferior temporal	Group	0.14	0.712	0.920
	Timepoint	2.89	0.089	0.275
	Group x timepoint	4.84	0.028	0.432
Isthmus cingulate	Group	3.39	0.066	0.387
	Timepoint	0.05	0.822	0.947
	Group x timepoint	0.37	0.543	0.850
Lateral occipital	Group	1.82	0.178	0.525
	Timepoint	4.18	0.041	0.199
	Group x timepoint	0.87	0.352	0.749
Lateral orbitofrontal	Group	0.03	0.864	0.920
	Timepoint	0.04	0.84	0.952
	Group x timepoint	0.15	0.701	0.948
Lingual	Group	0.13	0.719	0.920
	Timepoint	7.71	0.005	0.093
	Group x timepoint	2.98	0.085	0.479
Medial orbitofrontal	Group	0.80	0.372	0.752
	Timepoint	0.26	0.607	0.811

	Group x timepoint	1.00	0.317	0.743
Middle temporal	Group	4.62	0.032	0.304
	Timepoint	0.03	0.864	0.963
	Group x timepoint	1.66	0.198	0.651
Parahippocampal	Group	0.06	0.805	0.920
	Timepoint	1.77	0.183	0.443
	Group x timepoint	0.06	0.805	0.949
Paracentral	Group	2.06	0.151	0.513
	Timepoint	7.02	0.008	0.110
	Group x timepoint	0.005	0.945	0.980
Pars opercularis	Group	0.15	0.699	0.920
	Timepoint	0.68	0.410	0.716
	Group x timepoint	1.65	0.199	0.651
Pars orbitalis	Group	9.01	0.003	0.128
	Timepoint	4.76	0.029	0.180
	Group x timepoint	2.81	0.094	0.490
Pars triangularis	Group	1.20	0.273	0.688
	Timepoint	3.82	0.051	0.216
	Group x timepoint	8.82	0.003	0.102
Peri calcarine	Group	0.08	0.777	0.920
	Timepoint	2.41	0.121	0.334
	Group x timepoint	0.02	0.879	0.964
Post central	Group	0.04	0.841	0.920
	Timepoint	0.003	0.954	0.981
	Group x timepoint	0.50	0.479	0.814
Posterior cingulate	Group	0.55	0.458	0.752
	Timepoint	0.42	0.515	0.748
	Group x timepoint	1.53	0.217	0.657
Precentral	Group	0.45	0.500	0.752
	Timepoint	8.50	0.004	0.093
	Group x timepoint	1.43	0.232	0.657
Precuneus	Group	3.49	0.062	0.387
	Timepoint	0.57	0.452	0.748
	Group x timepoint	0.81	0.367	0.756
Rostral anterior cingulate	Group	0.05	0.822	0.920
	Timepoint	0.24	0.622	0.811

	Group x timepoint	0.54	0.464	0.809
Rostral middle frontal	Group	2.52	0.113	0.479
	Timepoint	1.47	0.225	0.487
	Group x timepoint	4.30	0.038	0.432
Superior frontal	Group	1.01	0.314	0.712
	Timepoint	3.41	0.065	0.232
	Group x timepoint	0.33	0.565	0.850
Superior parietal	Group	1.05	0.305	0.712
	Timepoint	4.44	0.035	0.199
	Group x timepoint	0.31	0.575	0.850
Superior temporal	Group	0.04	0.846	0.920
	Timepoint	0.15	0.696	0.845
	Group x timepoint	0.07	0.787	0.949
Supra marginal	Group	0.01	0.916	0.929
	Timepoint	3.26	0.071	0.235
	Group x timepoint	2.02	0.155	0.651
Frontal pole	Group	0.51	0.477	0.752
	Timepoint	0.51	0.476	0.748
	Group x timepoint	0.00	0.979	0.980
Temporal pole	Group	1.56	0.212	0.595
	Timepoint	0.19	0.664	0.836
	Group x timepoint	1.13	0.287	0.710
Transverse temporal	Group	0.56	0.454	0.752
	Timepoint	0.01	0.907	0.963
	Group x timepoint	0.27	0.605	0.875
Insula	Group	3.19	0.074	0.387
	Timepoint	0.68	0.411	0.716
	Group x timepoint	0.39	0.530	0.850

<footnote>

Type III tests of fixed effects were obtained from longitudinal mixed-effects models. The group effect tests overall LP–FT differences within the longitudinal model, timepoint effect tests baseline-to-follow-up changes, and group-by-timepoint effect tests whether longitudinal changes differed between the LP and FT children. Models included group, timepoint, and group-by-timepoint as fixed effects, with participant ID, family ID, and data collection site as random intercepts. Twin/triplet status and MRI scanner information were included as fixed-effect covariates. For neuroimaging outcomes, intracranial volume was included as a timepoint-specific covariate. F values are Type III F

statistics obtained from longitudinal mixed-effects models. Raw and FDR-adjusted p values are shown. FDR correction was applied within the corresponding outcome family. FDR-adjusted $p < 0.05$ was considered statistically significant. LP, late preterm; FT, full term; FDR, false discovery rate; MRI, magnetic resonance imaging.

Table S6. Complete Type III longitudinal mixed-effects model results for cortical surface area measures

Regions	Effect	F	raw_p	FDR_p
Banks of superior temporal sulcus	Group	5.47	0.019	0.202
	Timepoint	0.70	0.401	0.978
	Group x timepoint	5.15	0.023	0.940
Caudal anterior cingulate	Group	0.01	0.908	0.949
	Timepoint	4.52	0.033	0.569
	Group x timepoint	0.05	0.823	0.940
Caudal middle frontal	Group	0.01	0.903	0.949
	Timepoint	0.11	0.745	0.978
	Group x timepoint	0.004	0.95	0.965
Cuneus	Group	0.23	0.635	0.839
	Timepoint	0.14	0.706	0.978
	Group x timepoint	0.91	0.34	0.940
Entorhinal	Group	2.03	0.154	0.349
	Timepoint	1.42	0.233	0.934
	Group x timepoint	0.18	0.671	0.940
Fusiform	Group	7.86	0.005	0.115
	Timepoint	0.92	0.336	0.978
	Group x timepoint	1.03	0.310	0.940
Inferior parietal	Group	3.85	0.05	0.211
	Timepoint	0.33	0.568	0.978
	Group x timepoint	2.85	0.091	0.940
Inferior temporal	Group	0.81	0.367	0.589
	Timepoint	0.25	0.618	0.978
	Group x timepoint	0.08	0.781	0.940
Isthmus cingulate	Group	0.75	0.388	0.589
	Timepoint	0.29	0.593	0.978
	Group x timepoint	1.23	0.267	0.940
Lateral occipital	Group	0.00	0.99	0.990
	Timepoint	0.46	0.497	0.978
	Group x timepoint	1.03	0.309	0.940
Lateral orbitofrontal	Group	0.19	0.662	0.849
	Timepoint	3.83	0.05	0.685
	Group x timepoint	2.28	0.131	0.940

Lingual	Group	1.63	0.202	0.391
	Timepoint	1.69	0.193	0.923
	Group x timepoint	0.59	0.444	0.940
Medial orbitofrontal	Group	4.32	0.038	0.211
	Timepoint	2.46	0.117	0.853
	Group x timepoint	0.83	0.364	0.940
Middle temporal	Group	2.08	0.15	0.349
	Timepoint	0.26	0.612	0.978
	Group x timepoint	1.51	0.22	0.940
Parahippocampal	Group	0.01	0.941	0.965
	Timepoint	2.72	0.099	0.853
	Group x timepoint	0.38	0.535	0.940
Paracentral	Group	1.59	0.207	0.391
	Timepoint	0.07	0.798	0.978
	Group x timepoint	0.04	0.844	0.940
Pars opercularis	Group	2.61	0.106	0.301
	Timepoint	0.12	0.733	0.978
	Group x timepoint	0.80	0.371	0.940
Pars orbitalis	Group	1.73	0.189	0.378
	Timepoint	2.62	0.105	0.853
	Group x timepoint	0.21	0.643	0.940
Pars triangularis	Group	4.11	0.043	0.211
	Timepoint	0.46	0.499	0.978
	Group x timepoint	0.49	0.483	0.940
Peri calcarine	Group	0.004	0.951	0.965
	Timepoint	0.002	0.968	0.978
	Group x timepoint	0.10	0.754	0.940
Post central	Group	9.37	0.002	0.075
	Timepoint	0.83	0.363	0.978
	Group x timepoint	0.02	0.878	0.940
Posterior cingulate	Group	1.83	0.177	0.368
	Timepoint	1.05	0.305	0.978
	Group x timepoint	3.07	0.080	0.940
Precentral	Group	1.90	0.168	0.368
	Timepoint	1.93	0.164	0.914
	Group x timepoint	0.21	0.643	0.940

Precuneus	Group	2.53	0.112	0.305
	Timepoint	0.06	0.800	0.978
	Group x timepoint	0.28	0.599	0.940
Rostral anterior cingulate	Group	6.09	0.014	0.202
	Timepoint	0.28	0.594	0.978
	Group x timepoint	0.51	0.473	0.940
Rostral middle frontal	Group	0.05	0.817	0.903
	Timepoint	0.00	0.978	0.978
	Group x timepoint	0.03	0.870	0.940
Superior frontal	Group	0.73	0.393	0.589
	Timepoint	0.11	0.736	0.978
	Group x timepoint	0.09	0.759	0.940
Superior parietal	Group	0.50	0.48	0.680
	Timepoint	0.02	0.901	0.978
	Group x timepoint	0.88	0.349	0.940
Superior temporal	Group	0.04	0.846	0.914
	Timepoint	7.16	0.007	0.509
	Group x timepoint	0.53	0.467	0.940
Supra marginal	Group	1.81	0.179	0.368
	Timepoint	3.20	0.074	0.833
	Group x timepoint	0.23	0.635	0.940
Frontal pole	Group	0.11	0.738	0.878
	Timepoint	0.07	0.798	0.978
	Group x timepoint	0.97	0.325	0.940
Temporal pole	Group	0.63	0.428	0.619
	Timepoint	0.83	0.364	0.978
	Group x timepoint	0.37	0.544	0.940
Transverse temporal	Group	0.05	0.824	0.903
	Timepoint	1.09	0.298	0.978
	Group x timepoint	0.42	0.515	0.940
Insula	Group	3.42	0.064	0.230
	Timepoint	0.04	0.834	0.978
	Group x timepoint	1.36	0.243	0.940
Right				
Banks of superior temporal sulcus	Group	3.53	0.06	0.230
	Timepoint	0.86	0.352	0.978

	Group x timepoint	0.06	0.802	0.940
Caudal anterior cingulate	Group	2.32	0.128	0.321
	Timepoint	0.23	0.634	0.978
	Group x timepoint	0.62	0.432	0.940
Caudal middle frontal	Group	0.12	0.733	0.878
	Timepoint	0.56	0.453	0.978
	Group x timepoint	0.03	0.864	0.940
Cuneus	Group	0.71	0.398	0.589
	Timepoint	0.01	0.940	0.978
	Group x timepoint	0.02	0.884	0.940
Entorhinal	Group	2.25	0.134	0.325
	Timepoint	0.05	0.820	0.978
	Group x timepoint	1.90	0.168	0.940
Fusiform	Group	5.35	0.021	0.202
	Timepoint	0.001	0.973	0.978
	Group x timepoint	0.28	0.594	0.940
Inferior parietal	Group	4.06	0.044	0.211
	Timepoint	0.003	0.954	0.978
	Group x timepoint	0.09	0.765	0.940
Inferior temporal	Group	1.46	0.228	0.407
	Timepoint	0.01	0.912	0.978
	Group x timepoint	0.04	0.841	0.940
Isthmus cingulate	Group	1.52	0.217	0.400
	Timepoint	0.11	0.739	0.978
	Group x timepoint	0.02	0.887	0.940
Lateral occipital	Group	0.12	0.734	0.878
	Timepoint	0.40	0.528	0.978
	Group x timepoint	1.08	0.298	0.940
Lateral orbitofrontal	Group	2.83	0.092	0.300
	Timepoint	0.52	0.469	0.978
	Group x timepoint	0.76	0.383	0.940
Lingual	Group	0.10	0.749	0.878
	Timepoint	0.10	0.756	0.978
	Group x timepoint	0.88	0.349	0.940
Medial orbitofrontal	Group	3.51	0.061	0.230
	Timepoint	2.35	0.125	0.853

	Group x timepoint	0.49	0.484	0.940
Middle temporal	Group	5.80	0.016	0.202
	Timepoint	0.24	0.625	0.978
	Group x timepoint	1.31	0.252	0.940
Parahippocampal	Group	2.65	0.104	0.301
	Timepoint	0.41	0.522	0.978
	Group x timepoint	1.18	0.278	0.940
Paracentral	Group	0.78	0.379	0.589
	Timepoint	0.33	0.567	0.978
	Group x timepoint	1.66	0.198	0.940
Pars opercularis	Group	4.30	0.038	0.211
	Timepoint	0.005	0.946	0.978
	Group x timepoint	0.16	0.689	0.940
Pars orbitalis	Group	4.96	0.026	0.211
	Timepoint	0.003	0.959	0.978
	Group x timepoint	2.25	0.134	0.940
Pars triangularis	Group	3.94	0.047	0.211
	Timepoint	1.88	0.17	0.914
	Group x timepoint	2.44	0.119	0.940
Peri calcarine	Group	0.22	0.642	0.839
	Timepoint	0.03	0.866	0.978
	Group x timepoint	0.001	0.969	0.969
Post central	Group	2.33	0.127	0.321
	Timepoint	0.04	0.84	0.978
	Group x timepoint	0.22	0.639	0.940
Posterior cingulate	Group	1.01	0.314	0.548
	Timepoint	0.001	0.97	0.978
	Group x timepoint	4.63	0.031	0.940
Precentral	Group	0.14	0.709	0.878
	Timepoint	0.50	0.477	0.978
	Group x timepoint	0.13	0.722	0.940
Precuneus	Group	0.89	0.346	0.588
	Timepoint	4.72	0.03	0.569
	Group x timepoint	0.07	0.795	0.940
Rostral anterior cingulate	Group	4.78	0.029	0.211
	Timepoint	1.84	0.175	0.914

	Group x timepoint	0.02	0.899	0.940
Rostral middle frontal	Group	0.09	0.768	0.885
	Timepoint	0.05	0.815	0.978
	Group x timepoint	0.06	0.805	0.940
Superior frontal	Group	0.36	0.549	0.761
	Timepoint	0.03	0.871	0.978
	Group x timepoint	0.46	0.497	0.940
Superior parietal	Group	3.24	0.072	0.244
	Timepoint	0.17	0.681	0.978
	Group x timepoint	0.15	0.702	0.940
Superior temporal	Group	0.05	0.815	0.903
	Timepoint	0.61	0.435	0.978
	Group x timepoint	1.18	0.278	0.940
Supra marginal	Group	9.53	0.002	0.075
	Timepoint	1.62	0.204	0.923
	Group x timepoint	0.75	0.386	0.940
Frontal pole	Group	2.71	0.099	0.301
	Timepoint	0.54	0.463	0.978
	Group x timepoint	0.004	0.951	0.965
Temporal pole	Group	0.29	0.587	0.799
	Timepoint	1.49	0.222	0.934
	Group x timepoint	0.16	0.689	0.940
Transverse temporal	Group	0.72	0.396	0.589
	Timepoint	5.19	0.023	0.569
	Group x timepoint	1.00	0.317	0.940
Insula	Group	4.24	0.039	0.211
	Timepoint	0.08	0.779	0.978
	Group x timepoint	0.10	0.758	0.940

<footnote>

Type III tests of fixed effects were obtained from longitudinal mixed-effects models. The group effect tests overall LP–FT differences within the longitudinal model, timepoint effect tests baseline-to-follow-up changes, and group-by-timepoint effect tests whether longitudinal changes differed between the LP and FT children. Models included group, timepoint, and group-by-timepoint as fixed effects, with participant ID, family ID, and data collection site as random intercepts. Twin/triplet status and MRI scanner information were included as fixed-effect covariates. For neuroimaging outcomes, intracranial volume was included as a timepoint-specific covariate. F values are Type III F statistics obtained from longitudinal mixed-effects models. Raw and FDR-adjusted p values are

shown. FDR correction was applied within the corresponding outcome family. FDR-adjusted $p < 0.05$ was considered statistically significant. LP, late preterm; FT, full term; FDR, false discovery rate; MRI, magnetic resonance imaging.

Table S7. DTI

Table S7-1. Complete Type III longitudinal mixed-effects model results for white matter tract FA metrics

White matter tracts	Effect	F	raw_p	FDR_p
Rt. Fornix	Group	2.42	0.120	0.467
	Timepoint	7.19	0.007	**0.029**
	Group x timepoint	7.13	0.008	**0.046**
Lt. Fornix	Group	0.00	0.983	0.993
	Timepoint	12.81	<0.001	**0.003**
	Group x timepoint	6.86	0.009	**0.046**
Rt. Cingulate cingulum	Group	0.07	0.789	0.973
	Timepoint	10.68	0.001	**0.008**
	Group x timepoint	0.62	0.433	0.606
Lt. Cingulate cingulum	Group	0.65	0.419	0.771
	Timepoint	13.14	<0.001	**0.003**
	Group x timepoint	1.14	0.286	0.435
Rt. Parahippocampal cingulum	Group	1.01	0.315	0.771
	Timepoint	6.34	0.012	**0.036**
	Group x timepoint	0.37	0.543	0.656
Lt. Parahippocampal cingulum	Group	1.02	0.313	0.771
	Timepoint	8.54	0.003	**0.020**
	Group x timepoint	0.11	0.739	0.784
Rt. Corticospinal/pyramidal	Group	0.06	0.811	0.973
	Timepoint	0.33	0.564	0.705
	Group x timepoint	0.001	0.977	0.977
Lt. Corticospinal/pyramidal	Group	0.66	0.417	0.771
	Timepoint	0.54	0.463	0.648
	Group x timepoint	1.76	0.184	0.323
Rt. Anterior thalamic radiations	Group	0.84	0.36	0.771
	Timepoint	27.66	<0.001	**<0.001**
	Group x timepoint	2.01	0.156	0.322
Lt. Anterior thalamic radiations	Group	0.21	0.646	0.911
	Timepoint	5.74	0.017	**0.041**
	Group x timepoint	17.48	<0.001	**0.001**
Rt. Uncinate	Group	0.74	0.39	0.771
	Timepoint	0.07	0.793	0.816
	Group x timepoint	1.76	0.184	0.323

Lt. Uncinate	Group	0.50	0.479	0.799
	Timepoint	0.65	0.419	0.624
	Group x timepoint	9.70	0.002	**0.022**
Rt. Inferior longitudinal fasciculus	Group	10.9	<0.001	**0.034**
	Timepoint	0.25	0.618	0.721
	Group x timepoint	2.45	0.117	0.293
Lt. Inferior longitudinal fasciculus	Group	6.62	0.01	0.164
	Timepoint	0.38	0.536	0.695
	Group x timepoint	6.50	0.011	**0.046**
Rt. Inferior fronto-occipital fasciculus	Group	1.26	0.262	0.771
	Timepoint	5.82	0.016	**0.041**
	Group x timepoint	0.05	0.818	0.842
Lt. Inferior fronto-occipital fasciculus	Group	0.07	0.798	0.973
	Timepoint	1.71	0.191	0.371
	Group x timepoint	6.48	0.011	**0.046**
Forceps major	Group	0.20	0.651	0.911
	Timepoint	7.54	0.006	**0.026**
	Group x timepoint	0.25	0.617	0.696
Forceps minor	Group	0.04	0.834	0.973
	Timepoint	7.80	0.005	**0.026**
	Group x timepoint	3.82	0.051	0.136
Corpus callosum	Group	0.00	0.981	0.993
	Timepoint	0.40	0.53	0.695
	Group x timepoint	4.41	0.036	0.114
Rt. Superior longitudinal fasciculus	Group	0.24	0.621	0.911
	Timepoint	0.63	0.428	0.624
	Group x timepoint	1.80	0.179	0.323
Lt. Superior longitudinal fasciculus	Group	0.31	0.578	0.911
	Timepoint	2.40	0.121	0.265
	Group x timepoint	0.52	0.471	0.626
Rt. Temporal superior longitudinal fasciculus	Group	0.78	0.379	0.771
	Timepoint	4.06	0.044	0.102
	Group x timepoint	1.23	0.267	0.425
Lt. Temporal superior longitudinal fasciculus	Group	0.66	0.416	0.771
	Timepoint	1.55	0.214	0.394
	Group x timepoint	0.49	0.483	0.626

Rt. Parietal superior longitudinal fasciculus	Group	0.14	0.712	0.958
	Timepoint	0.14	0.707	0.773
	Group x timepoint	1.69	0.194	0.324
Lt. Parietal superior longitudinal fasciculus	Group	0.01	0.931	0.993
	Timepoint	1.93	0.165	0.340
	Group x timepoint	0.90	0.342	0.499
Rt. Superior corticostriate	Group	5.23	0.022	0.195
	Timepoint	1.36	0.244	0.427
	Group x timepoint	6.47	0.011	**0.046**
Lt. Superior corticostriate	Group	3.44	0.064	0.351
	Timepoint	1.16	0.282	0.470
	Group x timepoint	2.15	0.142	0.312
Rt. Superior corticostriate-frontal cortex	Group	0.50	0.478	0.799
	Timepoint	14.39	<0.001	**0.003**
	Group x timepoint	2.18	0.14	0.312
Lt. Superior corticostriate-frontal cortex	Group	1.23	0.268	0.771
	Timepoint	6.54	0.011	**0.036**
	Group x timepoint	4.21	0.04	0.118
Rt. Superior corticostriate-parietal cortex	Group	6.03	0.014	0.164
	Timepoint	0.01	0.905	0.905
	Group x timepoint	6.33	0.012	**0.046**
Lt. Superior corticostriate-parietal cortex	Group	3.29	0.07	0.351
	Timepoint	0.09	0.768	0.815
	Group x timepoint	0.38	0.54	0.656
Rt. Striatal inferior frontal cortex	Group	0.00	0.993	0.993
	Timepoint	1.07	0.301	0.480
	Group x timepoint	0.26	0.607	0.696
Lt. Striatal inferior frontal cortex	Group	0.01	0.931	0.993
	Timepoint	0.17	0.683	0.771
	Group x timepoint	13.95	<0.001	**0.003**
Rt. Inferior frontal superior frontal cortex	Group	2.52	0.113	0.467
	Timepoint	0.27	0.600	0.721
	Group x timepoint	0.20	0.658	0.720
Lt. Inferior frontal superior frontal cortex	Group	3.28	0.07	0.351
	Timepoint	6.29	0.012	**0.036**
	Group x timepoint	5.67	0.017	0.060

<footnote>

Type III tests of fixed effects were obtained from longitudinal mixed-effects models. The group effect tests overall LP–FT differences within the longitudinal model, timepoint effect tests baseline-to-follow-up changes, and group-by-timepoint effect tests whether longitudinal changes differed between the LP and FT children. Models included group, timepoint, and group-by-timepoint as fixed effects, with participant ID, family ID, and data collection site as random intercepts. Twin/triplet status and MRI scanner information were included as fixed-effect covariates. For neuroimaging outcomes, intracranial volume was included as a timepoint-specific covariate. F values are Type III F statistics from longitudinal mixed-effects models. Raw and FDR-adjusted p values are shown. FDR correction was applied within the corresponding outcome family. FDR-adjusted $p < 0.05$ was considered statistically significant. Bold values indicate these significant results. Estimates are not shown in this table; post hoc simple-effects results for significant group-by-timepoint interactions are provided separately. DTI, diffusion tensor imaging; FA, fractional anisotropy; LP, late preterm; FT, full term; FDR, false discovery rate; Lt., left; Rt., right; MRI, magnetic resonance imaging.

Table S7-2. Complete Type III longitudinal mixed-effects model results for white matter tract MD metrics

White matter tracts	Effect	F	raw_p	FDR_p
Rt. Fornix	Group	1.41	0.235	0.357
	Timepoint	14.12	<0.001	**<0.001**
	Group x timepoint	3.12	0.077	0.205
Lt. Fornix	Group	1.72	0.19	0.317
	Timepoint	13.97	<0.001	**<0.001**
	Group x timepoint	0.39	0.531	0.744
Rt. Cingulate cingulum	Group	0.07	0.798	0.798
	Timepoint	3.51	0.061	0.069
	Group x timepoint	0.80	0.370	0.648
Lt. Cingulate cingulum	Group	0.33	0.565	0.659
	Timepoint	0.11	0.744	0.766
	Group x timepoint	7.91	0.005	**0.039**
Rt. Parahippocampal cingulum	Group	0.12	0.725	0.753
	Timepoint	37.71	<0.001	**<0.001**
	Group x timepoint	0.49	0.486	0.708
Lt. Parahippocampal cingulum	Group	3.73	0.054	0.188
	Timepoint	11.24	<0.001	**0.001**
	Group x timepoint	0.06	0.809	0.967
Rt. Corticospinal/pyramidal	Group	6.47	0.011	0.089
	Timepoint	16.67	<0.001	**<0.001**
	Group x timepoint	0.06	0.805	0.967
Lt. Corticospinal/pyramidal	Group	5.16	0.023	0.101
	Timepoint	19.41	<0.001	**<0.001**
	Group x timepoint	0.62	0.431	0.672
Rt. Anterior thalamic radiations	Group	2.18	0.139	0.276
	Timepoint	0.09	0.770	0.770
	Group x timepoint	0.05	0.826	0.967
Lt. Anterior thalamic radiations	Group	5.69	0.017	0.100
	Timepoint	0.69	0.405	0.430
	Group x timepoint	1.84	0.175	0.354
Rt. Uncinate	Group	0.12	0.732	0.753
	Timepoint	9.24	0.002	**0.003**
	Group x timepoint	6.18	0.013	0.053
Lt. Uncinate	Group	2.38	0.123	0.276

	Timepoint	14.77	<0.001	**<0.001**
	Group x timepoint	0.74	0.389	0.648
Rt. Inferior longitudinal fasciculus	Group	5.38	0.02	0.101
	Timepoint	18.45	<0.001	**<0.001**
	Group x timepoint	7.85	0.005	**0.039**
Lt. Inferior longitudinal fasciculus	Group	14.53	<0.001	**0.005**
	Timepoint	7.70	0.006	**0.007**
	Group x timepoint	0.03	0.859	0.967
Rt. Inferior fronto-occipital fasciculus	Group	1.91	0.167	0.293
	Timepoint	8.44	0.004	**0.005**
	Group x timepoint	8.75	0.003	**0.039**
Lt. Inferior fronto-occipital fasciculus	Group	3.92	0.048	0.186
	Timepoint	11.69	<0.001	**0.001**
	Group x timepoint	0.01	0.915	0.967
Forceps major	Group	0.96	0.327	0.408
	Timepoint	23.72	<0.001	**<0.001**
	Group x timepoint	2.23	0.136	0.297
Forceps minor	Group	0.29	0.591	0.668
	Timepoint	7.25	0.007	**0.009**
	Group x timepoint	0.59	0.442	0.672
Corpus callosum	Group	0.59	0.443	0.535
	Timepoint	18.51	<0.001	**<0.001**
	Group x timepoint	0.02	0.875	0.967
Rt. Superior longitudinal fasciculus	Group	2.22	0.137	0.276
	Timepoint	53.84	<0.001	**<0.001**
	Group x timepoint	4.88	0.027	0.086
Lt. Superior longitudinal fasciculus	Group	1.06	0.302	0.392
	Timepoint	29.04	<0.001	**<0.001**
	Group x timepoint	0.02	0.891	0.967
Rt. Temporal superior longitudinal fasciculus	Group	6.21	0.013	0.089
	Timepoint	47.48	<0.001	**<0.001**
	Group x timepoint	1.78	0.182	0.354
Lt. Temporal superior longitudinal fasciculus	Group	1.97	0.161	0.293
	Timepoint	25.77	<0.001	**<0.001**
	Group x timepoint	0.002	0.967	0.967
Rt. Parietal superior longitudinal fasciculus	Group	1.19	0.276	0.374

	Timepoint	53.91	<0.001	**<0.001**
	Group x timepoint	6.1	0.014	0.053
Lt. Parietal superior longitudinal fasciculus	Group	0.18	0.671	0.734
	Timepoint	34.7	<0.001	**<0.001**
	Group x timepoint	0.04	0.838	0.967
Rt. Superior corticostriate	Group	1.18	0.278	0.374
	Timepoint	37.42	<0.001	**<0.001**
	Group x timepoint	7.71	0.006	**0.039**
Lt. Superior corticostriate	Group	2.45	0.117	0.276
	Timepoint	35.92	<0.001	**<0.001**
	Group x timepoint	1.02	0.312	0.575
Rt. Superior corticostriate-frontal cortex	Group	3.23	0.072	0.211
	Timepoint	19.22	<0.001	**<0.001**
	Group x timepoint	4.10	0.043	0.126
Lt. Superior corticostriate-frontal cortex	Group	3.39	0.066	0.209
	Timepoint	19.84	<0.001	**<0.001**
	Group x timepoint	2.78	0.096	0.223
Rt. Superior corticostriate-parietal cortex	Group	1.19	0.275	0.374
	Timepoint	39.22	<0.001	**<0.001**
	Group x timepoint	8.17	0.004	**0.039**
Lt. Superior corticostriate-parietal cortex	Group	1.62	0.203	0.323
	Timepoint	43.64	<0.001	**<0.001**
	Group x timepoint	0.005	0.945	0.967
Rt. Striatal inferior frontal cortex	Group	2.15	0.142	0.276
	Timepoint	4.99	0.026	**0.030**
	Group x timepoint	3.03	0.082	0.205
Lt. Striatal inferior frontal cortex	Group	6.91	0.009	0.089
	Timepoint	2.03	0.154	0.169
	Group x timepoint	7.28	0.007	**0.041**
Rt. Inferior frontal superior frontal cortex	Group	2.94	0.087	0.233
	Timepoint	9.71	0.002	**0.003**
	Group x timepoint	5.45	0.02	0.069
Lt. Inferior frontal superior frontal cortex	Group	6.86	0.009	0.089
	Timepoint	6.55	0.01	**0.013**
	Group x timepoint	6.29	0.012	0.053

<footnote>

Type III tests of fixed effects were obtained from longitudinal mixed-effects models. The group effect tests overall LP–FT differences within the longitudinal model, timepoint effect tests baseline-to-follow-up changes, and group-by-timepoint effect tests whether longitudinal changes differed between the LP and FT children. Models included group, timepoint, and group-by-timepoint as fixed effects, with participant ID, family ID, and data collection site as random intercepts. Twin/triplet status and MRI scanner information were included as fixed-effect covariates. For neuroimaging outcomes, intracranial volume was included as a timepoint-specific covariate. F values are Type III F statistics from longitudinal mixed-effects models. Raw and FDR-adjusted p values are shown. FDR correction was applied within the corresponding outcome family. FDR-adjusted $p < 0.05$ was considered statistically significant. Bold values indicate these significant results. Estimates are not shown in this table; post hoc simple-effects results for significant group x timepoint interactions are provided separately. DTI, diffusion tensor imaging; MD, mean diffusivity; MRI, magnetic resonance imaging; LP, late preterm; FT, full term; FDR, false discovery rate; Lt., left; Rt., right.

Table S7-3. Complete Type III longitudinal mixed-effects model results for DTI cortical ROIs FA metrics

Cortical ROIs	Effect	F	raw_p	FDR_p
Left				
Banks of superior temporal sulcus	Group	0.04	0.849	0.902
	Timepoint	1.07	0.301	0.417
	Group x timepoint	5.02	0.025	0.201
Caudal anterior cingulate	Group	2.33	0.127	0.261
	Timepoint	4.99	0.026	0.053
	Group x timepoint	4.99	0.025	0.201
Caudal middle frontal	Group	2.76	0.097	0.252
	Timepoint	0.90	0.342	0.465
	Group x timepoint	0.30	0.584	0.932
Cuneus	Group	0.56	0.456	0.620
	Timepoint	18.12	<0.001	**<0.001**
	Group x timepoint	1.03	0.309	0.658
Entorhinal	Group	1.26	0.261	0.395
	Timepoint	5.64	0.018	**0.039**
	Group x timepoint	1.16	0.282	0.658
Fusiform	Group	4.27	0.039	0.126
	Timepoint	0.06	0.800	0.892
	Group x timepoint	0.06	0.809	0.948
Inferior parietal	Group	0.94	0.333	0.482
	Timepoint	0.002	0.967	0.973
	Group x timepoint	0.03	0.862	0.961
Inferior temporal	Group	0.42	0.518	0.665
	Timepoint	1.57	0.210	0.311
	Group x timepoint	1.73	0.189	0.487
Isthmus cingulate	Group	0.01	0.926	0.954
	Timepoint	6.47	0.011	**0.029**
	Group x timepoint	6.33	0.012	0.201
Lateral occipital	Group	4.69	0.030	0.109
	Timepoint	0.11	0.746	0.845
	Group x timepoint	0.38	0.537	0.909
Lateral orbitofrontal	Group	1.52	0.217	0.395
	Timepoint	0.001	0.973	0.973
	Group x timepoint	4.73	0.03	0.201

Lingual	Group	11.49	<0.001	**0.007**
	Timepoint	4.46	0.035	0.068
	Group x timepoint	1.78	0.182	0.487
Medial orbitofrontal	Group	0.14	0.708	0.816
	Timepoint	2.18	0.14	0.232
	Group x timepoint	2.11	0.147	0.475
Middle temporal	Group	7.57	0.006	**0.034**
	Timepoint	1.10	0.293	0.416
	Group x timepoint	2.35	0.125	0.426
Parahippocampal	Group	0.08	0.776	0.879
	Timepoint	0.001	0.969	0.973
	Group x timepoint	6.47	0.011	0.201
Paracentral	Group	1.36	0.244	0.395
	Timepoint	6.91	0.009	**0.023**
	Group x timepoint	6.37	0.012	0.201
Pars opercularis	Group	5.24	0.022	0.094
	Timepoint	7.48	0.006	**0.019**
	Group x timepoint	2.40	0.121	0.426
Pars orbitalis	Group	2.55	0.111	0.261
	Timepoint	0.02	0.894	0.965
	Group x timepoint	1.39	0.239	0.580
Pars triangularis	Group	2.88	0.09	0.245
	Timepoint	7.30	0.007	**0.020**
	Group x timepoint	4.98	0.026	0.201
Peri calcarine	Group	13.58	<0.001	**0.004**
	Timepoint	17.28	<0.001	**<0.001**
	Group x timepoint	0.04	0.833	0.948
Post central	Group	5.26	0.022	0.094
	Timepoint	5.20	0.023	**0.048**
	Group x timepoint	0.003	0.954	0.983
Posterior cingulate	Group	0.37	0.543	0.671
	Timepoint	0.70	0.402	0.536
	Group x timepoint	0.16	0.685	0.937
Precentral	Group	0.24	0.626	0.746
	Timepoint	5.60	0.018	**0.039**
	Group x timepoint	0.06	0.812	0.948

Precuneus	Group	9.05	0.003	**0.018**
	Timepoint	1.77	0.184	0.278
	Group x timepoint	3.45	0.063	0.287
Rostral anterior cingulate	Group	18.78	<0.001	**0.001**
	Timepoint	2.07	0.150	0.244
	Group x timepoint	18.68	<0.001	**0.001**
Rostral middle frontal	Group	1.75	0.185	0.360
	Timepoint	0.22	0.638	0.787
	Group x timepoint	0.15	0.702	0.937
Superior frontal	Group	1.44	0.231	0.395
	Timepoint	0.02	0.883	0.965
	Group x timepoint	2.82	0.093	0.373
Superior parietal	Group	0.59	0.442	0.614
	Timepoint	2.77	0.096	0.164
	Group x timepoint	0.12	0.730	0.937
Superior temporal	Group	12.11	<0.001	**0.007**
	Timepoint	0.12	0.729	0.840
	Group x timepoint	4.84	0.028	0.201
Supra marginal	Group	7.83	0.005	**0.032**
	Timepoint	6.41	0.011	**0.029**
	Group x timepoint	0.006	0.940	0.983
Frontal pole	Group	0.04	0.843	0.902
	Timepoint	14.16	<0.001	**<0.001**
	Group x timepoint	0.68	0.411	0.770
Temporal pole	Group	1.68	0.194	0.367
	Timepoint	3.07	0.080	0.139
	Group x timepoint	4.01	0.045	0.248
Transverse temporal	Group	9.43	0.002	**0.016**
	Timepoint	11.49	<0.001	**0.003**
	Group x timepoint	0.12	0.730	0.937
Insula	Group	1.28	0.257	0.395
	Timepoint	0.13	0.718	0.840
	Group x timepoint	1.11	0.293	0.658
Right				
Banks of superior temporal sulcus	Group	0.99	0.321	0.474
	Timepoint	9.42	0.002	**0.007**

	Group x timepoint	0.29	0.59	0.932
Caudal anterior cingulate	Group	0.33	0.566	0.687
	Timepoint	4.21	0.04	0.074
	Group x timepoint	0.08	0.781	0.948
Caudal middle frontal	Group	5.71	0.017	0.082
	Timepoint	31.83	<0.001	**<0.001**
	Group x timepoint	5.49	0.019	0.201
Cuneus	Group	0.003	0.956	0.969
	Timepoint	39.09	<0.001	**<0.001**
	Group x timepoint	4.36	0.037	0.228
Entorhinal	Group	0.04	0.839	0.902
	Timepoint	15.3	<0.001	**<0.001**
	Group x timepoint	0.04	0.836	0.948
Fusiform	Group	1.34	0.247	0.395
	Timepoint	0.21	0.648	0.787
	Group x timepoint	0.15	0.697	0.937
Inferior parietal	Group	5.08	0.024	0.097
	Timepoint	19.23	<0.001	**<0.001**
	Group x timepoint	0.24	0.627	0.935
Inferior temporal	Group	3.94	0.047	0.146
	Timepoint	12.07	<0.001	**0.002**
	Group x timepoint	0.13	0.716	0.937
Isthmus cingulate	Group	0.04	0.833	0.902
	Timepoint	0.48	0.487	0.628
	Group x timepoint	0.23	0.632	0.935
Lateral orbitofrontal	Group	2.62	0.105	0.261
	Timepoint	6.20	0.013	**0.031**
	Group x timepoint	0.00	0.977	0.983
Lateral orbitofrontal	Group	1.41	0.236	0.395
	Timepoint	4.89	0.027	0.054
	Group x timepoint	0.05	0.819	0.948
Lingual	Group	13.47	<0.001	**0.004**
	Timepoint	1.87	0.172	0.265
	Group x timepoint	0.76	0.383	0.743
Medial orbitofrontal	Group	0.49	0.486	0.648
	Timepoint	10.57	0.001	**0.004**

	Group x timepoint	0.003	0.959	0.983
Middle temporal	Group	14.10	<0.001	**0.004**
	Timepoint	0.48	0.489	0.628
	Group x timepoint	1.73	0.188	0.487
Parahippocampal	Group	0.002	0.969	0.969
	Timepoint	0.01	0.926	0.973
	Group x timepoint	3.15	0.076	0.324
Paracentral	Group	1.94	0.163	0.327
	Timepoint	0.18	0.674	0.804
	Group x timepoint	1.94	0.163	0.487
Pars opercularis	Group	2.39	0.122	0.261
	Timepoint	17.46	<0.001	**<0.001**
	Group x timepoint	0.00	0.983	0.983
Pars orbitalis	Group	3.34	0.068	0.200
	Timepoint	1.94	0.164	0.259
	Group x timepoint	0.09	0.759	0.948
Pars triangularis	Group	4.50	0.034	0.115
	Timepoint	13.71	<0.001	**<0.001**
	Group x timepoint	0.62	0.430	0.770
Peri calcarine	Group	11.54	<0.001	**0.007**
	Timepoint	5.80	0.016	**0.038**
	Group x timepoint	0.36	0.548	0.909
Post central	Group	2.37	0.124	0.261
	Timepoint	19.66	<0.001	**<0.001**
	Group x timepoint	0.62	0.430	0.770
Posterior cingulate	Group	0.75	0.386	0.547
	Timepoint	9.00	0.003	**0.008**
	Group x timepoint	3.81	0.051	0.248
Precentral	Group	0.46	0.496	0.649
	Timepoint	14.63	<0.001	**<0.001**
	Group x timepoint	0.43	0.514	0.896
Precuneus	Group	3.04	0.081	0.230
	Timepoint	0.28	0.596	0.750
	Group x timepoint	0.01	0.91	0.983
Rostral anterior cingulate	Group	2.52	0.112	0.261
	Timepoint	10.52	0.001	**0.004**

	Group x timepoint	0.99	0.320	0.659
Rostral middle frontal	Group	9.53	0.002	**0.016**
	Timepoint	4.23	0.04	0.074
	Group x timepoint	2.57	0.109	0.412
Superior frontal	Group	0.38	0.538	0.671
	Timepoint	25.16	<0.001	**<0.001**
	Group x timepoint	0.17	0.679	0.937
Superior parietal	Group	0.02	0.880	0.921
	Timepoint	12.15	<0.001	**0.002**
	Group x timepoint	0.01	0.920	0.983
Superior temporal	Group	7.14	0.008	**0.039**
	Timepoint	1.49	0.222	0.321
	Group x timepoint	3.93	0.048	0.248
Supra marginal	Group	0.18	0.674	0.790
	Timepoint	30	<0.001	**<0.001**
	Group x timepoint	1.69	0.193	0.487
Frontal pole	Group	2.38	0.123	0.261
	Timepoint	17.73	<0.001	**<0.001**
	Group x timepoint	0.79	0.375	0.743
Temporal pole	Group	1.33	0.249	0.395
	Timepoint	23.93	<0.001	**<0.001**
	Group x timepoint	1.04	0.307	0.658
Transverse temporal	Group	1.29	0.256	0.395
	Timepoint	3.48	0.062	0.111
	Group x timepoint	0.24	0.625	0.935
Insula	Group	4.84	0.028	0.105
	Timepoint	0.01	0.935	0.973
	Group x timepoint	1.78	0.182	0.487

<footnote>

Type III tests of fixed effects were obtained from longitudinal mixed-effects models. The group effect tests overall LP–FT differences within the longitudinal model, timepoint effect tests baseline-to-follow-up changes, and group-by-timepoint effect tests whether longitudinal changes differed between the LP and FT children. Models included group, timepoint, and group-by-timepoint as fixed effects, with participant ID, family ID, and data collection site as random intercepts. Twin/triplet status and MRI scanner information were included as fixed-effect covariates. For neuroimaging outcomes, intracranial volume was included as a timepoint-specific covariate. F values are Type III F

statistics obtained from longitudinal mixed-effects models. Raw and FDR-adjusted p values are shown. FDR correction was applied within the corresponding outcome family. FDR-adjusted $p < 0.05$ was considered statistically significant. Bold values indicate these significant results. Estimates are not shown in this table; post hoc simple-effects results for significant group-by-timepoint interactions are provided separately. DTI, diffusion tensor imaging; ROI, region of interest; FA, fractional anisotropy; LP, late preterm; FT, full term; FDR, false discovery rate; Lt., left; Rt., right; MRI, magnetic resonance imaging.

Table S7-4. Complete Type III longitudinal mixed-effects model results for DTI cortical ROIs MD metrics

ROIs	Effect	F	raw_p	FDR_p
Left				
Banks of superior temporal sulcus	Group	4.51	0.034	0.294
	Timepoint	27.57	<0.001	**<0.001**
	Group x timepoint	0.31	0.576	0.781
Caudal anterior cingulate	Group	0.003	0.956	0.976
	Timepoint	0.00	0.988	0.988
	Group x timepoint	0.67	0.414	0.762
Caudal middle frontal	Group	1.71	0.191	0.525
	Timepoint	3.72	0.054	0.065
	Group x timepoint	0.43	0.511	0.781
Cuneus	Group	2.09	0.148	0.480
	Timepoint	18.8	<0.001	**<0.001**
	Group x timepoint	3.30	0.069	0.215
Entorhinal	Group	3.44	0.064	0.380
	Timepoint	5.94	0.015	**0.020**
	Group x timepoint	0.00	0.99	0.990
Fusiform	Group	9.42	0.002	0.073
	Timepoint	17.96	<0.001	**<0.001**
	Group x timepoint	0.31	0.579	0.781
Inferior parietal	Group	6.14	0.013	0.225
	Timepoint	28.48	<0.001	**<0.001**
	Group x timepoint	0.3	0.587	0.781
Inferior temporal	Group	2.47	0.116	0.466
	Timepoint	10.07	0.002	**0.002**
	Group x timepoint	0.28	0.597	0.781
Isthmus cingulate	Group	0.00	0.98	0.980
	Timepoint	14.38	<0.001	**<0.001**
	Group x timepoint	1.86	0.173	0.391
Lateral occipital	Group	1.58	0.209	0.525
	Timepoint	30.71	<0.001	**<0.001**
	Group x timepoint	3.14	0.077	0.217
Lateral orbitofrontal	Group	1.59	0.207	0.525
	Timepoint	2.79	0.095	0.113

	Group x timepoint	0.33	0.564	0.781
Lingual	Group	7.39	0.007	0.149
	Timepoint	62.7	<0.001	**<0.001**
	Group x timepoint	0.08	0.782	0.883
Medial orbitofrontal	Group	0.01	0.928	0.971
	Timepoint	0.14	0.709	0.731
	Group x timepoint	2.33	0.127	0.321
Middle temporal	Group	9.58	0.002	0.073
	Timepoint	10.15	0.001	**0.002**
	Group x timepoint	0.51	0.476	0.781
Parahippocampal	Group	4.92	0.027	0.294
	Timepoint	12.65	<0.001	**<0.001**
	Group x timepoint	1.94	0.164	0.384
Paracentral	Group	0.04	0.838	0.971
	Timepoint	22.04	<0.001	**<0.001**
	Group x timepoint	0.52	0.470	0.781
Pars opercularis	Group	3.95	0.047	0.319
	Timepoint	32.07	<0.001	**<0.001**
	Group x timepoint	3.29	0.070	0.215
Pars orbitalis	Group	0.03	0.863	0.971
	Timepoint	7.22	0.007	**0.010**
	Group x timepoint	2.32	0.127	0.321
Pars triangularis	Group	3.10	0.078	0.380
	Timepoint	15.39	<0.001	**<0.001**
	Group x timepoint	0.005	0.945	0.986
Peri calcarine	Group	2.63	0.105	0.466
	Timepoint	31.45	<0.001	**<0.001**
	Group x timepoint	3.56	0.059	0.215
Post central	Group	1.04	0.307	0.612
	Timepoint	35.3	<0.001	**<0.001**
	Group x timepoint	0.09	0.763	0.879
Posterior cingulate	Group	0.45	0.502	0.775
	Timepoint	10.64	0.001	**0.002**
	Group x timepoint	6.63	0.01	0.076
Precentral	Group	0.62	0.430	0.697
	Timepoint	15.11	<0.001	**<0.001**

	Group x timepoint	0.14	0.711	0.868
Precuneus	Group	1.12	0.291	0.612
	Timepoint	22.58	<0.001	**<0.001**
	Group x timepoint	0.81	0.369	0.697
Rostral anterior cingulate	Group	0.94	0.333	0.612
	Timepoint	0.004	0.949	0.964
	Group x timepoint	1.11	0.292	0.568
Rostral middle frontal	Group	0.80	0.370	0.629
	Timepoint	4.64	0.031	**0.039**
	Group x timepoint	0.23	0.629	0.792
Superior frontal	Group	1.96	0.162	0.500
	Timepoint	2.25	0.133	0.157
	Group x timepoint	1.15	0.285	0.568
Superior parietal	Group	1.15	0.284	0.612
	Timepoint	23.01	<0.001	**<0.001**
	Group x timepoint	0.10	0.749	0.878
Superior temporal	Group	1.71	0.192	0.525
	Timepoint	16.34	<0.001	**<0.001**
	Group x timepoint	0.003	0.957	0.986
Supra marginal	Group	4.47	0.035	0.294
	Timepoint	47.07	<0.001	**<0.001**
	Group x timepoint	0.57	0.452	0.781
Frontal pole	Group	0.19	0.664	0.871
	Timepoint	0.16	0.692	0.724
	Group x timepoint	0.34	0.557	0.781
Temporal pole	Group	0.12	0.73	0.895
	Timepoint	15.35	<0.001	**<0.001**
	Group x timepoint	0.54	0.463	0.781
Transverse temporal	Group	0.82	0.365	0.629
	Timepoint	26.34	<0.001	**<0.001**
	Group x timepoint	3.47	0.063	0.215
Insula	Group	0.97	0.325	0.612
	Timepoint	22.70	<0.001	**<0.001**
	Group x timepoint	3.58	0.059	0.215
Right				
Banks of superior temporal sulcus	Group	1.01	0.315	0.612

	Timepoint	20.03	<0.001	**<0.001**
	Group x timepoint	4.67	0.031	0.149
Caudal anterior cingulate	Group	0.22	0.638	0.868
	Timepoint	0.61	0.434	0.483
	Group x timepoint	3.16	0.076	0.217
Caudal middle frontal	Group	3.20	0.074	0.380
	Timepoint	23.42	<0.001	**<0.001**
	Group x timepoint	1.40	0.237	0.488
Cuneus	Group	0.42	0.518	0.783
	Timepoint	41.62	<0.001	**<0.001**
	Group x timepoint	0.05	0.821	0.895
Entorhinal	Group	2.55	0.111	0.466
	Timepoint	4.78	0.029	**0.037**
	Group x timepoint	0.001	0.975	0.990
Fusiform	Group	4.15	0.042	0.314
	Timepoint	23.32	<0.001	**<0.001**
	Group x timepoint	2.80	0.094	0.256
Inferior parietal	Group	3.23	0.072	0.380
	Timepoint	43.87	<0.001	**<0.001**
	Group x timepoint	5.72	0.017	0.095
Inferior temporal	Group	0.01	0.928	0.971
	Timepoint	37.76	<0.001	**<0.001**
	Group x timepoint	5.79	0.016	0.095
Isthmus cingulate	Group	1.07	0.301	0.612
	Timepoint	20.85	<0.001	**<0.001**
	Group x timepoint	1.57	0.210	0.460
Lateral occipital	Group	0.02	0.898	0.971
	Timepoint	43.97	<0.001	**<0.001**
	Group x timepoint	0.32	0.575	0.781
Lateral orbitofrontal	Group	2.16	0.142	0.480
	Timepoint	0.49	0.485	0.517
	Group x timepoint	3.42	0.064	0.215
Lingual	Group	5.30	0.021	0.291
	Timepoint	60.95	<0.001	**<0.001**
	Group x timepoint	1.43	0.232	0.488
Medial orbitofrontal	Group	2.35	0.125	0.472

	Timepoint	0.48	0.487	0.517
	Group x timepoint	0.02	0.886	0.941
Middle temporal	Group	1.19	0.275	0.612
	Timepoint	16.70	<0.001	**<0.001**
	Group x timepoint	8.44	0.004	**0.031**
Parahippocampal	Group	0.82	0.364	0.629
	Timepoint	31.28	<0.001	**<0.001**
	Group x timepoint	2.00	0.157	0.382
Paracentral	Group	0.35	0.557	0.788
	Timepoint	26.84	<0.001	**<0.001**
	Group x timepoint	0.07	0.792	0.883
Pars opercularis	Group	0.11	0.737	0.895
	Timepoint	23.60	<0.001	**<0.001**
	Group x timepoint	15.23	<0.001	**0.007**
Pars orbitalis	Group	0.14	0.708	0.895
	Timepoint	1.98	0.159	0.184
	Group x timepoint	5.42	0.02	0.104
Pars triangularis	Group	0.02	0.902	0.971
	Timepoint	11.37	<0.001	**0.001**
	Group x timepoint	11.32	<0.001	**0.018**
Peri calcarine	Group	1.42	0.233	0.546
	Timepoint	50.31	<0.001	**<0.001**
	Group x timepoint	0.24	0.625	0.792
Post central	Group	0.36	0.551	0.788
	Timepoint	37.65	<0.001	**<0.001**
	Group x timepoint	9.66	0.002	**0.027**
Posterior cingulate	Group	0.002	0.962	0.976
	Timepoint	6.87	0.009	**0.012**
	Group x timepoint	0.13	0.72	0.868
Precentral	Group	0.06	0.809	0.965
	Timepoint	27.00	<0.001	**<0.001**
	Group x timepoint	12.30	<0.001	**0.016**
Precuneus	Group	1.47	0.226	0.546
	Timepoint	28.86	<0.001	**<0.001**
	Group x timepoint	0.12	0.727	0.868
Rostral anterior cingulate	Group	0.37	0.543	0.788

	Timepoint	0.59	0.441	0.483
	Group x timepoint	0.31	0.578	0.781
Rostral middle frontal	Group	0.22	0.635	0.868
	Timepoint	10.52	0.001	**0.002**
	Group x timepoint	4.47	0.034	0.156
Superior frontal	Group	1.60	0.206	0.525
	Timepoint	9.35	0.002	**0.003**
	Group x timepoint	0.47	0.495	0.781
Superior parietal	Group	0.19	0.666	0.871
	Timepoint	40.61	<0.001	**<0.001**
	Group x timepoint	3.54	0.06	0.215
Superior temporal	Group	2.14	0.143	0.480
	Timepoint	22.79	<0.001	**<0.001**
	Group x timepoint	8.96	0.003	**0.027**
Supra marginal	Group	0.49	0.483	0.764
	Timepoint	57.28	<0.001	**<0.001**
	Group x timepoint	9.07	0.003	**0.027**
Frontal pole	Group	0.65	0.421	0.697
	Timepoint	1.26	0.262	0.297
	Group x timepoint	0.39	0.533	0.781
Temporal pole	Group	0.02	0.901	0.971
	Timepoint	3.75	0.053	0.065
	Group x timepoint	0.05	0.829	0.895
Transverse temporal	Group	0.13	0.717	0.895
	Timepoint	30.44	<0.001	**<0.001**
	Group x timepoint	6.12	0.013	0.091
Insula	Group	0.03	0.861	0.971
	Timepoint	5.86	0.015	**0.020**
	Group x timepoint	9.49	0.002	**0.027**

<footnote>

Type III tests of fixed effects were obtained from longitudinal mixed-effects models. The group effect tests overall LP–FT differences within the longitudinal model, timepoint effect tests baseline-to-follow-up changes, and group-by-timepoint effect tests whether longitudinal changes differed between the LP and FT children. Models included group, timepoint, and group-by-timepoint as fixed effects, with participant ID, family ID, and data collection site as random intercepts. Twin/triplet status and MRI scanner information were included as fixed-effect covariates. For neuroimaging outcomes, intracranial volume was included as a timepoint-specific covariate. F values are Type III F

statistics obtained from longitudinal mixed-effects models. Raw and FDR-adjusted p values are shown. FDR correction was applied within the corresponding outcome family. FDR-adjusted $p < 0.05$ was considered statistically significant. Bold values indicate these significant results. Estimates are not shown in this table; post hoc simple-effects results for significant group-by-timepoint interactions are provided separately. DTI, diffusion tensor imaging; ROI, region of interest; MRI, magnetic resonance imaging; MD, mean diffusivity; LP, late preterm; FT, full term; FDR, false discovery rate; Lt., left; Rt., right.

Table S7-5. Complete Type III longitudinal mixed-effects model results for DTI subcortical ROIs FA metrics

ROIs	Effect	F	raw_p	FDR_p
Left				
Thalamus	Group	1.83	0.176	0.224
	Timepoint	3.30	0.069	0.069
	Group x timepoint	1.99	0.158	0.184
Caudate	Group	7.21	0.007	**0.015**
	Timepoint	35.51	<0.001	**<0.001**
	Group x timepoint	1.39	0.239	0.239
Putamen	Group	8.45	0.004	**0.010**
	Timepoint	20.65	<0.001	**<0.001**
	Group x timepoint	14.60	<0.001	**0.002**
Pallidum	Group	5.62	0.018	**0.031**
	Timepoint	34.22	<0.001	**<0.001**
	Group x timepoint	8.03	0.005	**0.013**
Hippocampus	Group	11.10	<0.001	**0.006**
	Timepoint	3.70	0.054	0.064
	Group x timepoint	12.76	<0.001	**0.002**
Amygdala	Group	2.44	0.119	0.166
	Timepoint	9.69	0.002	**0.002**
	Group x timepoint	10.37	0.001	**0.005**
Accumbens area	Group	0.01	0.918	0.918
	Timepoint	19.74	<0.001	**<0.001**
	Group x timepoint	3.09	0.079	0.101
Right				
Thalamus	Group	0.60	0.439	0.473
	Timepoint	21.57	<0.001	**<0.001**
	Group x timepoint	7.21	0.007	**0.017**
Caudate	Group	3.7	0.055	0.085
	Timepoint	86.31	<0.001	**<0.001**
	Group x timepoint	1.65	0.200	0.215
Putamen	Group	9.52	0.002	**0.009**
	Timepoint	94.38	<0.001	**<0.001**
	Group x timepoint	4.41	0.036	0.063
Pallidum	Group	11.04	<0.001	**0.006**
	Timepoint	66.52	<0.001	**<0.001**

	Group x timepoint	12.26	<0.001	**0.002**
Hippocampus	Group	9.10	0.003	**0.009**
	Timepoint	13.31	<0.001	**<0.001**
	Group x timepoint	4.39	0.036	0.063
Amygdala	Group	8.18	0.004	**0.010**
	Timepoint	3.30	0.069	0.069
	Group x timepoint	3.87	0.049	0.077
Accumbens area	Group	0.91	0.34	0.397
	Timepoint	10.81	0.001	**0.001**
	Group x timepoint	3.69	0.055	0.077

<footnote>

Type III tests of fixed effects were obtained from longitudinal mixed-effects models. The group effect tests overall LP–FT differences within the longitudinal model, timepoint effect tests baseline-to-follow-up changes, and group-by-timepoint effect tests whether longitudinal changes differed between the LP and FT children. Models included group, timepoint, and group-by-timepoint as fixed effects, with participant ID, family ID, and data collection site as random intercepts. Twin/triplet status and MRI scanner information were included as fixed-effect covariates. For neuroimaging outcomes, intracranial volume was included as a timepoint-specific covariate. F values are Type III F statistics obtained from longitudinal mixed-effects models. Raw and FDR-adjusted p values are shown. FDR correction was applied within the corresponding outcome family. FDR-adjusted $p < 0.05$ was considered statistically significant. Bold values indicate these significant results. Estimates are not shown in this table; post hoc simple-effects results for significant group-by-timepoint interactions are provided separately. DTI, diffusion tensor imaging; ROI, region of interest; FA, fractional anisotropy; MRI, magnetic resonance imaging; LP, late preterm; FT, full term; FDR, false discovery rate; Lt., left; Rt., right.

Table S7-6. Complete Type III longitudinal mixed-effects model results for DTI subcortical ROIs MD metrics

ROIs	Effect	F	raw_p	FDR_p
Left				
Thalamus	Group	1.57	0.211	0.591
	Timepoint	20.42	<0.001	**<0.001**
	Group x timepoint	1.37	0.242	0.377
Caudate	Group	0.04	0.85	0.927
	Timepoint	2.47	0.116	0.147
	Group x timepoint	0.40	0.525	0.613
Putamen	Group	0.66	0.416	0.684
	Timepoint	23.48	<0.001	**<0.001**
	Group x timepoint	2.20	0.138	0.373
Pallidum	Group	7.25	0.007	0.050
	Timepoint	3.10	0.078	0.109
	Group x timepoint	1.95	0.163	0.373
Hippocampus	Group	0.69	0.408	0.684
	Timepoint	24.71	<0.001	**<0.001**
	Group x timepoint	10.84	<0.001	**0.007**
Amygdala	Group	0.12	0.732	0.927
	Timepoint	39.63	<0.001	**<0.001**
	Group x timepoint	0.04	0.848	0.848
Accumbens area	Group	0.60	0.44	0.684
	Timepoint	0.24	0.622	0.622
	Group x timepoint	0.78	0.378	0.524
Right				
Thalamus	Group	0.48	0.490	0.686
	Timepoint	24.07	<0.001	**<0.001**
	Group x timepoint	1.40	0.237	0.377
Caudate	Group	4.31	0.038	0.177
	Timepoint	10.86	<0.001	**0.002**
	Group x timepoint	4.71	0.030	0.140
Putamen	Group	0.01	0.927	0.927
	Timepoint	8.31	0.004	**0.006**
	Group x timepoint	3.55	0.06	0.209
Pallidum	Group	10.19	0.001	**0.020**
	Timepoint	0.74	0.390	0.421

	Group x timepoint	0.22	0.642	0.691
Hippocampus	Group	0.03	0.865	0.927
	Timepoint	36.59	<0.001	**<0.001**
	Group x timepoint	14.20	<0.001	**0.002**
Amygdala	Group	2.33	0.127	0.444
	Timepoint	36.36	<0.001	**<0.001**
	Group x timepoint	1.75	0.186	0.373
Accumbens area	Group	0.89	0.344	0.684
	Timepoint	1.23	0.268	0.313
	Group x timepoint	0.67	0.412	0.524

<footnote>

Type III tests of fixed effects were obtained from longitudinal mixed-effects models. The group effect tests overall LP–FT differences within the longitudinal model, timepoint effect tests baseline-to-follow-up changes, and group-by-timepoint effect tests whether longitudinal changes differed between the LP and FT children. Models included group, timepoint, and group-by-timepoint as fixed effects, with participant ID, family ID, and data collection site as random intercepts. Twin/triplet status and MRI scanner information were included as fixed-effect covariates. For neuroimaging outcomes, intracranial volume was included as a timepoint-specific covariate. F values are Type III F statistics obtained from longitudinal mixed-effects models. Raw and FDR-adjusted p values are shown. FDR correction was applied within the corresponding outcome family. FDR-adjusted $p < 0.05$ was considered statistically significant. Bold values indicate these significant results. Estimates are not shown in this table; post hoc simple-effects results for significant group-by-timepoint interactions are provided separately. DTI, diffusion tensor imaging; ROI, region of interest; MD, mean diffusivity; MRI, magnetic resonance imaging; LP, late preterm; FT, full term; FDR, false discovery rate; Lt., left; Rt., right.

Table S8. Complete Type III longitudinal mixed-effects model results for rs-fMRI within network connectivity

Connectivity	Effect	F	raw_p	FDR_p
AN	Group	1.94	0.164	0.657
	Timepoint	857.17	<0.001	**<0.001**
	Group x timepoint	1.94	0.163	0.313
CON	Group	0.12	0.727	0.882
	Timepoint	938.24	<0.001	**<0.001**
	Group x timepoint	5.90	0.015	0.097
CPN	Group	5.41	0.02	0.241
	Timepoint	674.27	<0.001	**<0.001**
	Group x timepoint	1.78	0.183	0.313
DMN	Group	0.06	0.808	0.882
	Timepoint	1027.63	<0.001	**<0.001**
	Group x timepoint	2.30	0.13	0.311
DAN	Group	1.17	0.279	0.836
	Timepoint	940.32	<0.001	**<0.001**
	Group x timepoint	2.58	0.108	0.311
FRN	Group	0.06	0.807	0.882
	Timepoint	555.07	<0.001	**<0.001**
	Group x timepoint	0.36	0.547	0.729
RTN	Group	0.00	0.988	0.988
	Timepoint	757.54	<0.001	**<0.001**
	Group x timepoint	1.14	0.285	0.428
SMHN	Group	2.26	0.133	0.657
	Timepoint	715.22	<0.001	**<0.001**
	Group x timepoint	0.09	0.76	0.830
SMMN	Group	0.07	0.789	0.882
	Timepoint	559.04	<0.001	**<0.001**
	Group x timepoint	0.09	0.759	0.830
SN	Group	0.39	0.534	0.882
	Timepoint	690.49	<0.001	**<0.001**
	Group x timepoint	0.03	0.868	0.868
VAN	Group	0.15	0.698	0.882
	Timepoint	731.91	<0.001	**<0.001**
	Group x timepoint	2.95	0.086	0.311
VN	Group	0.26	0.612	0.882

	Timepoint	928.9	<0.001	**<0.001**
	Group x timepoint	5.78	0.016	0.097

<footnote>

Type III tests of fixed effects were obtained from longitudinal mixed-effects models. The group effect tests overall LP–FT differences within the longitudinal model, timepoint effect tests baseline-to-follow-up changes, and group-by-timepoint effect tests whether longitudinal changes differed between the LP and FT children. Models included group, timepoint, and group-by-timepoint as fixed effects, with participant ID, family ID, and data collection site as random intercepts. Twin/triplet status and MRI scanner information were included as fixed-effect covariates. For neuroimaging outcomes, intracranial volume was included as a timepoint-specific covariate. F values are Type III F statistics obtained from longitudinal mixed-effects models. Raw and FDR-adjusted p values are shown. FDR correction was applied within the corresponding outcome family. FDR-adjusted $p < 0.05$ was considered statistically significant. Bold values indicate these significant results. LP, late preterm; FT, full term; FDR, false discovery rate; AN, auditory network; CON, cingulo-opercular network; CPN, cingulo-parietal network; DMN, default mode network; DAN, dorsal attention network; FRN, fronto-parietal network; fMRI, functional magnetic resonance imaging; RTN, retrosplenial temporal network; SMHN, sensorimotor hand network; SMMN, sensorimotor mouth network; SN, salience network; VAN, ventral attention network; VN, visual network.

Table S9. Post hoc simple-effects analyses for DTI metrics with significant group-by-timepoint interactions

	Tracts/ROIs	Comparison	Level	Estimate (95% CI)	FDR-*p*
White matter tracts FA	Lt. Fornix	LP-FT within each timepoint	baseline	-0.049 (-0.122, 0.024)	0.274
		LP-FT within each timepoint	follow-up	0.052 (-0.037, 0.142)	0.302
		Follow-up – baseline within each group	FT	-0.176 (-0.239, -0.113)	**< 0.0001**
		Follow-up – baseline within each group	LP	-0.074 (-0.167, 0.018)	0.208
	Lt. Anterior thalamic radiations	LP-FT within each timepoint	baseline	-0.058 (-0.125, 0.008)	0.195
		LP-FT within each timepoint	follow-up	0.090 (0.007, 0.172)	0.099
		Follow-up – baseline within each group	FT	-0.151 (-0.209, -0.094)	**< 0.0001**
		Follow-up – baseline within each group	LP	-0.004 (-0.088, 0.081)	0.934
	Lt. Uncinate	LP-FT within each timepoint	baseline	-0.079 (-0.150, -0.009)	0.099
		LP-FT within each timepoint	follow-up	0.028 (-0.057, 0.112)	0.556
		Follow-up – baseline within each group	FT	-0.082 (-0.142, -0.022)	**0.034**
		Follow-up – baseline within each group	LP	0.025 (-0.060, 0.110)	0.721
	Lt. Inferior longitudinal fasciculus	LP-FT within each timepoint	baseline	0.055 (-0.020, 0.130)	0.247
		LP-FT within each timepoint	follow-up	0.137 (0.050, 0.225)	**0.025**
		Follow-up – baseline within each group	FT	-0.069 (-0.133, -0.006)	0.093
		Follow-up – baseline within each group	LP	0.013 (-0.072, 0.098)	0.924
	Lt. Inferior fronto-occipital fasciculus	LP-FT within each timepoint	baseline	-0.030 (-0.098, 0.038)	0.439
		LP-FT within each timepoint	follow-up	0.050 (-0.031, 0.131)	0.290
		Follow-up – baseline within each group	FT	0.004	0.934

				(-0.053, 0.062)	
		Follow-up – baseline within each group	LP	0.084<br>(0.005, 0.163)	0.096
	Lt. Striatal inferior frontal cortex	LP-FT within each timepoint	baseline	-0.062<br>(-0.129, 0.005)	0.175
		LP-FT within each timepoint	follow-up	0.068<br>(-0.014, 0.150)	0.208
		Follow-up – baseline within each group	FT	-0.052<br>(-0.110, 0.005)	0.148
		Follow-up – baseline within each group	LP	0.078<br>(-0.006, 0.162)	0.148
	Rt. Fornix	LP-FT within each timepoint	baseline	0.007<br>(-0.060, 0.075)	0.833
		LP-FT within each timepoint	follow-up	0.102<br>(0.019, 0.184)	0.072
		Follow-up – baseline within each group	FT	-0.132<br>(-0.191, -0.074)	**< 0.0001**
		Follow-up – baseline within each group	LP	-0.038<br>(-0.123, 0.047)	0.526
	Rt. Superior corticostriate	LP-FT within each timepoint	baseline	0.051<br>(-0.017, 0.120)	0.247
		LP-FT within each timepoint	follow-up	0.120<br>(0.042, 0.198)	**0.025**
		Follow-up – baseline within each group	FT	-0.032<br>(-0.089, 0.026)	0.455
		Follow-up – baseline within each group	LP	0.037<br>(-0.037, 0.112)	0.495
	Rt. Superior corticostriate-parietal cortex only	LP-FT within each timepoint	baseline	0.063<br>(-0.003, 0.129)	0.123
		LP-FT within each timepoint	follow-up	0.132<br>(0.056, 0.208)	**0.010**
		Follow-up – baseline within each group	FT	-0.031<br>(-0.088, 0.026)	0.463
		Follow-up – baseline within each group	LP	0.038<br>(-0.037, 0.112)	0.481
White matter tracts MD	Lt. Cingulate cingulum	LP-FT within each timepoint	baseline	-0.042<br>(-0.115, 0.031)	0.442
		LP-FT within each timepoint	follow-up	0.091<br>(-0.004, 0.187)	0.123
		Follow-up – baseline within each group	FT	-0.073<br>(-0.138, -0.007)	**0.043**

	Follow-up – baseline within each group	LP	0.061 (-0.044, 0.165)	0.305
Lt. Striatal inferior frontal cortex	LP-FT within each timepoint	baseline	-0.031 (-0.097, 0.036)	0.528
	LP-FT within each timepoint	follow-up	-0.140 (-0.226, -0.054)	**0.008**
	Follow-up – baseline within each group	FT	0.011 (-0.047, 0.070)	0.700
	Follow-up – baseline within each group	LP	-0.098 (-0.190, -0.005)	0.051
Rt. Inferior longitudinal fasciculus	LP-FT within each timepoint	baseline	-0.135 (-0.209, -0.061)	**0.004**
	LP-FT within each timepoint	follow-up	-0.038 (-0.126, 0.050)	0.528
	Follow-up – baseline within each group	FT	-0.194 (-0.257, -0.132)	**< 0.0001**
	Follow-up – baseline within each group	LP	-0.097 (-0.184, -0.011)	**0.043**
Rt. Inferior fronto-occipital fasciculus	LP-FT within each timepoint	baseline	-0.106 (-0.178, -0.033)	**0.018**
	LP-FT within each timepoint	follow-up	0.003 (-0.085, 0.092)	0.855
	Follow-up – baseline within each group	FT	-0.155 (-0.216, -0.093)	**< 0.0001**
	Follow-up – baseline within each group	LP	-0.046 (-0.135, 0.043)	0.346
Rt. Superior corticostriate	LP-FT within each timepoint	baseline	-0.088 (-0.157, -0.020)	**0.028**
	LP-FT within each timepoint	follow-up	0.014 (-0.071, 0.098)	0.822
	Follow-up – baseline within each group	FT	-0.251 (-0.309, -0.192)	**< 0.0001**
	Follow-up – baseline within each group	LP	-0.149 (-0.235, -0.062)	**0.002**
Rt. Superior corticostriate-parietal cortex only	LP-FT within each timepoint	baseline	-0.089 (-0.159, -0.020)	**0.028**
	LP-FT within each timepoint	follow-up	0.014 (-0.071, 0.098)	0.822
	Follow-up – baseline within each group	FT	-0.257 (-0.316, -0.198)	**< 0.0001**
	Follow-up – baseline within each group	LP	-0.154	**0.001**

				(-0.240, -0.068)	
Cortical ROIs FA	Lt. Rostral anterior cingulate	LP-FT within each timepoint	baseline	0.069 (-0.003, 0.141)	0.059
		LP-FT within each timepoint	follow-up	0.242 (0.152, 0.332)	**< 0.0001**
		Follow-up – baseline within each group	FT	-0.142 (-0.207, -0.078)	**< 0.0001**
		Follow-up – baseline within each group	LP	0.030 (-0.065, 0.126)	0.531
Cortical ROIs MD	Rt. Middle temporal	LP-FT within each timepoint	baseline	-0.090 (-0.156, -0.023)	0.077
		LP-FT within each timepoint	follow-up	0.026 (-0.058, 0.110)	0.583
		Follow-up – baseline within each group	FT	-0.184 (-0.243, -0.126)	**< 0.0001**
		Follow-up – baseline within each group	LP	-0.068 (-0.158, 0.021)	0.152
	Rt. Pars opercularis	LP-FT within each timepoint	baseline	-0.061 (-0.126, 0.0085	0.138
		LP-FT within each timepoint	follow-up	0.089 (0.006, 0.172)	0.112
		Follow-up – baseline within each group	FT	-0.228 (-0.285, -0.170)	**< 0.0001**
		Follow-up – baseline within each group	LP	-0.077 (-0.165, 0.010)	0.102
	Rt. Pars triangularis	LP-FT within each timepoint	baseline	-0.069 (-0.133, -0.005)	0.112
		LP-FT within each timepoint	follow-up	0.068 (-0.015, 0.151)	0.155
		Follow-up – baseline within each group	FT	-0.174 (-0.231, -0.116)	**< 0.0001**
		Follow-up – baseline within each group	LP	-0.036 (-0.127, 0.054)	0.462
	Rt. Post central	LP-FT within each timepoint	baseline	-0.039 (-0.104, 0.025)	0.279
		LP-FT within each timepoint	follow-up	0.083 (0.000, 0.165)	0.132
		Follow-up – baseline within each group	FT	-0.255 (-0.312, -0.199)	**< 0.0001**
		Follow-up – baseline within each group	LP	-0.133 (-0.221, -0.045)	**0.005**

	Rt. Precentral	LP-FT within each timepoint	baseline	-0.062 (-0.127, 0.004)	0.138
		LP-FT within each timepoint	follow-up	0.050 (-0.029, 0.128)	0.279
		Follow-up – baseline within each group	FT	-0.210 (-0.265, -0.155)	**< 0.0001**
		Follow-up – baseline within each group	LP	-0.099 (-0.177, -0.021)	**0.019**
	Rt. Superior temporal	LP-FT within each timepoint	baseline	-0.003 (-0.066, 0.060)	0.923
		LP-FT within each timepoint	follow-up	0.105 (0.025, 0.184)	0.077
		Follow-up – baseline within each group	FT	-0.195 (-0.251, -0.140)	**< 0.0001**
		Follow-up – baseline within each group	LP	-0.088 (-0.170, -0.005)	0.050
	Rt. Superior marginal	LP-FT within each timepoint	baseline	-0.071 (-0.135, -0.008)	0.112
		LP-FT within each timepoint	follow-up	0.033 (-0.046, 0.111)	0.471
		Follow-up – baseline within each group	FT	-0.278 (-0.333, -0.223)	**< 0.0001**
		Follow-up – baseline within each group	LP	-0.174 (-0.255, -0.093)	**< 0.0001**
	Rt.Insula	LP-FT within each timepoint	baseline	-0.055 (-0.118, 0.007)	0.143
		LP-FT within each timepoint	follow-up	0.071 (-0.011, 0.154)	0.143
		Follow-up – baseline within each group	FT	-0.137 (-0.193, -0.081)	**< 0.0001**
		Follow-up – baseline within each group	LP	-0.010 (-0.101, 0.080)	0.821
Subcortical ROIs FA	Lt. Putamen	LP-FT within each timepoint	baseline	0.004 (-0.045, 0.053)	0.879
		LP-FT within each timepoint	follow-up	0.131 (0.065, 0.196)	**0.0003**
		Follow-up – baseline within each group	FT	-0.187 (-0.231, -0.142)	**< 0.0001**
		Follow-up – baseline within each group	LP	-0.060 (-0.133, 0.014)	0.134
	Lt. Pallidum	LP-FT within each timepoint	baseline	0.005	0.879

				(-0.056, 0.065)	
		LP-FT within each timepoint	follow-up	0.127 (0.044, 0.209)	**0.008**
		Follow-up – baseline within each group	FT	-0.261 (-0.317, -0.205)	< **0.0001**
		Follow-up – baseline within each group	LP	-0.139 (-0.234, -0.044)	**0.006**
	Lt. hippocampus	LP-FT within each timepoint	baseline	0.020 (-0.024, 0.064)	0.612
		LP-FT within each timepoint	follow-up	0.123 (0.065, 0.181)	**0.0003**
		Follow-up – baseline within each group	FT	-0.098 (-0.138, -0.058)	< **0.0001**
		Follow-up – baseline within each group	LP	0.005 (-0.059, 0.069)	0.879
	Lt. Amygdala	LP-FT within each timepoint	baseline	-0.015 (-0.072, 0.041)	0.795
		LP-FT within each timepoint	follow-up	0.104 (0.029, 0.178)	**0.015**
		Follow-up – baseline within each group	FT	-0.153 (-0.205, -0.101)	< **0.0001**
		Follow-up – baseline within each group	LP	-0.034 (-0.117, 0.049)	0.464
	Rt. Thalamus	LP-FT within each timepoint	baseline	-0.029 (-0.098, 0.040)	0.612
		LP-FT within each timepoint	follow-up	0.083 (-0.006, 0.171)	0.134
		Follow-up – baseline within each group	FT	-0.219 (-0.280, -0.158)	< **0.0001**
		Follow-up – baseline within each group	LP	-0.107 (-0.202, -0.012)	**0.036**
	Rt.Pallidum	LP-FT within each timepoint	baseline	0.012 (-0.042, 0.066)	0.795
		LP-FT within each timepoint	follow-up	0.152 (0.078, 0.226)	**0.0003**
		Follow-up – baseline within each group	FT	-0.318 (-0.368, -0.268)	< **0.0001**
		Follow-up – baseline within each group	LP	-0.178 (-0.264, -0.091)	**0.0001**
Subcortical ROIs MD	Lt. hippocampus	LP-FT within each timepoint	baseline	-0.093 (-0.156, -0.029)	**0.018**

	LP-FT within each timepoint	follow-up	0.038 (-0.044, 0.121)	0.363
	Follow-up – baseline within each group	FT	-0.230 (-0.287, -0.173)	**< 0.0001**
	Follow-up – baseline within each group	LP	-0.099 (-0.189, -0.009)	**0.031**
Rt. hippocampus	LP-FT within each timepoint	baseline	-0.088 (-0.154, -0.022)	**0.019**
	LP-FT within each timepoint	follow-up	0.075 (-0.013, 0.162)	0.124
	Follow-up – baseline within each group	FT	-0.292 (-0.351, -0.232)	**< 0.0001**
	Follow-up – baseline within each group	LP	-0.129 (-0.225, -0.033)	**0.011**

<footnote>

Post hoc simple-effects analyses were conducted only for DTI measures showing significant group-by-timepoint interactions after FDR correction. LP–FT contrasts indicate between-group differences at each timepoint. Follow-up–baseline contrasts indicate within-group longitudinal changes. Estimates and 95% confidence intervals are based on standardized outcomes from the longitudinal mixed-effects models. These analyses were considered descriptive and were used to characterize the direction of the significant interaction terms. FDR-adjusted $p < 0.05$ was considered statistically significant. Bold values indicate these significant results. DTI, diffusion tensor imaging; LP, late preterm; FT, full term; FDR, false discovery rate; ROI, region of interest; FA, fractional anisotropy; MD, mean diffusivity; CI, confidence interval; Lt, left; Rt, right.

Table S10. Attrition analyses for brain volume and DTI at 2-year follow-up

	Baseline N	Follow-up N	Overall retention n	LP retention n	FT retention n	χ^2 *p*	LP/FT SMD	Adjusted LP OR	OR *p*
Brain volume	10029	4721	47.2%	48.3%	46.9%	0.391	0.018	0.969	0.676
WMT-FA	9160	4105	44.8%	46.3%	44.6%	0.324	0.021	0.984	0.838
WMT-MD	9160	4105	44.8%	46.3%	44.6%	0.324	0.021	0.984	0.838
Cortical ROIs-FA	9159	4104	44.8%	46.3%	44.6%	0.322	0.021	0.985	0.840
Cortical ROIs-MD	9159	4104	44.8%	46.3%	44.6%	0.322	0.021	0.985	0.840
Subcortical ROIs-FA	9160	4105	44.8%	46.3%	44.6%	0.324	0.021	0.984	0.838
Subcortical ROIs-MD	9160	4105	44.8%	46.3%	44.6%	0.324	0.021	0.984	0.838

Completers were defined as participants with available data for the corresponding imaging domain at both baseline and the two-year follow-up. Non-completers had available baseline data but incomplete or unavailable follow-up data for that domain. The retention rate was calculated as the number of completers divided by the number of participants with available baseline data. Retention rates were compared between LP and FT children using chi-square tests. Adjusted odds ratios were obtained from multivariable logistic regression models predicting follow-up data availability from LP/FT group status and predefined baseline covariates. Standardized mean differences were used to compare baseline characteristics between completers and non-completers, with an absolute SMD ≥ 0.10 considered indicative of potential imbalance. DTI, diffusion tensor imaging; LP, late preterm; FT, full term; SMD, standard mean difference; OR, odds ratio; WMT, white matter tract; ROI, region of interest; FA, fractional anisotropy; MD, mean diffusivity; MRI, magnetic resonance imaging
